# Syndecan-4 Phosphorylation Is a Control Point for Integrin Recycling

**DOI:** 10.1016/j.devcel.2013.01.027

**Published:** 2013-03-11

**Authors:** Mark R. Morgan, Hellyeh Hamidi, Mark D. Bass, Stacey Warwood, Christoph Ballestrem, Martin J. Humphries

**Affiliations:** 1 Wellcome Trust Centre for Cell-Matrix Research, Faculty of Life Sciences, University of Manchester, Michael Smith Building, Oxford Road, Manchester M13 9PT, UK

## Abstract

Precise spatiotemporal coordination of integrin adhesion complex dynamics is essential for efficient cell migration. For cells adherent to fibronectin, differential engagement of α_5_β_1_ and α_V_β_3_ integrins is used to elicit changes in adhesion complex stability, mechanosensation, matrix assembly, and migration, but the mechanisms responsible for receptor regulation have remained largely obscure. We identify phosphorylation of the membrane-intercalated proteoglycan syndecan-4 as an essential switch controlling integrin recycling. Src phosphorylates syndecan-4 and, by driving syntenin binding, leads to suppression of Arf6 activity and recycling of α_V_β_3_ to the plasma membrane at the expense of α_5_β_1_. The resultant elevation in α_V_β_3_ engagement promotes stabilization of focal adhesions. Conversely, abrogation of syndecan-4 phosphorylation drives surface expression of α_5_β_1_, destabilizes adhesion complexes, and disrupts cell migration. These data identify the dynamic spatiotemporal regulation of Src-mediated syndecan-4 phosphorylation as an essential switch controlling integrin trafficking and adhesion dynamics to promote efficient cell migration.

## Graphical abstract

**Graphical Abstract:**

**Figure:**
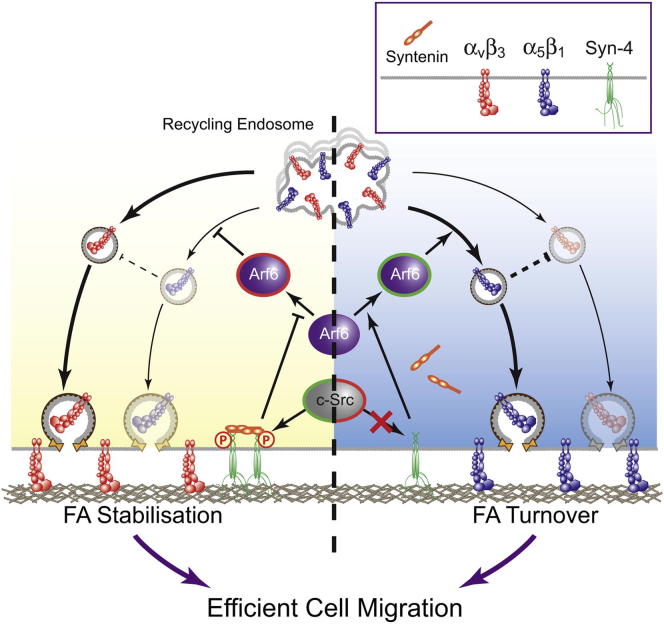

**Highlights:**

► c-Src phosphorylates syndecan-4 in response to extracellular stimuli ► Syndecan-4 phosphorylation and engagement regulate Arf6 activity ► Syndecan-4-mediated Arf6 activity regulates differential integrin recycling ► Syndecan-4-mediated integrin recycling controls FA dynamics and cell migration

## Teaser

Efficient cell migration requires dynamic coordination of cell-matrix interactions, in part through precise spatiotemporal control of integrin adhesion dynamics. Morgan et al. show that phosphorylation of the extracellular matrix receptor syndecan-4 acts as a molecular switch, differentially directing either α_5_β_1_ or α_V_β_3_ integrin recycling in the control of adhesion dynamics.

## Introduction

Haptotactic migration, in which cells are guided by direct interactions of adhesion receptors with extracellular matrix (ECM) fibers, is fundamental to tissue morphogenesis, homeostasis, and repair and for the pathogenesis of inflammatory and neoplastic diseases. Focal adhesions (FAs) are sites of cell-ECM integration where topological features of the ECM are interpreted. FAs contain clusters of integrin receptors and hundreds of cytoskeletal and signaling molecules. These complexes function as both physical links to the contractile cytoskeletal machinery and dynamic signaling nexuses. Crucially, efficient cell migration requires the precise spatial and temporal regulation of FA turnover and stabilization (Geiger et al., 2001; Ridley et al., 2003).

Engagement of different integrin heterodimers by the same ECM ligand elicits remarkably different cellular responses (Morgan et al., 2009). The fibronectin-binding integrins α_5_β_1_ and α_V_β_3_ exhibit distinct biomechanical, mechanoresponsive, and signaling properties that directly influence the dynamic interaction with the ECM and cell migration (Danen et al., 2002, 2005; Hu et al., 2007; Puklin-Faucher and Sheetz, 2009; Roca-Cusachs et al., 2009). It follows that, during cell migration in vivo, heterodimer-specific integrin localization at the cell-ECM interface must be tightly regulated. Intracellular trafficking pathways spatially and temporally segregate engagement of, and signaling from, specific integrin heterodimers, and accumulating evidence suggests that integrin recycling plays a key role in cell migration and disease progression (Caswell et al., 2009; Roberts et al., 2001; White et al., 2007). Thus, elucidating the precise mechanisms that control heterodimer-specific trafficking of integrins, and how this process modulates FA dynamics, is fundamental to understanding how cell migration is coordinated.

Syndecans are transmembrane heparan sulfate proteoglycans that act as receptors for ECM molecules and coreceptors for growth factors, cytokines, and morphogens (Alexopoulou et al., 2007; Morgan et al., 2007; Murakami et al., 2008). The fibronectin receptor syndecan-4 regulates GTPase activity and adhesive function to modulate cell migration (Bass et al., 2007a, 2007b, 2008; Dovas et al., 2006; Morgan et al., 2007; Woods et al., 1986). We have recently described a potential role for syndecan-4 in regulating integrin endocytosis (Bass et al., 2011), but the extent to which syndecans integrate extracellular and intracellular stimuli to directly regulate integrin function has otherwise not been investigated.

Here we demonstrate that syndecan-4 is the major control point that regulates integrin recycling to coordinate FA dynamics and cell migration. c-Src-mediated syndecan-4 phosphorylation is shown to regulate Arf6 activity, via modulation of syntenin binding, and acts as a molecular switch to determine directly whether α_5_β_1_ or α_V_β_3_ integrins are delivered to the membrane. Thus, we define a mechanism by which syndecan-4 engagement and signaling exquisitely controls integrin engagement to dictate FA stability and coordinate cell migration.

## Results

### Src Phosphorylates Syndecan-4

**Figure 1:**
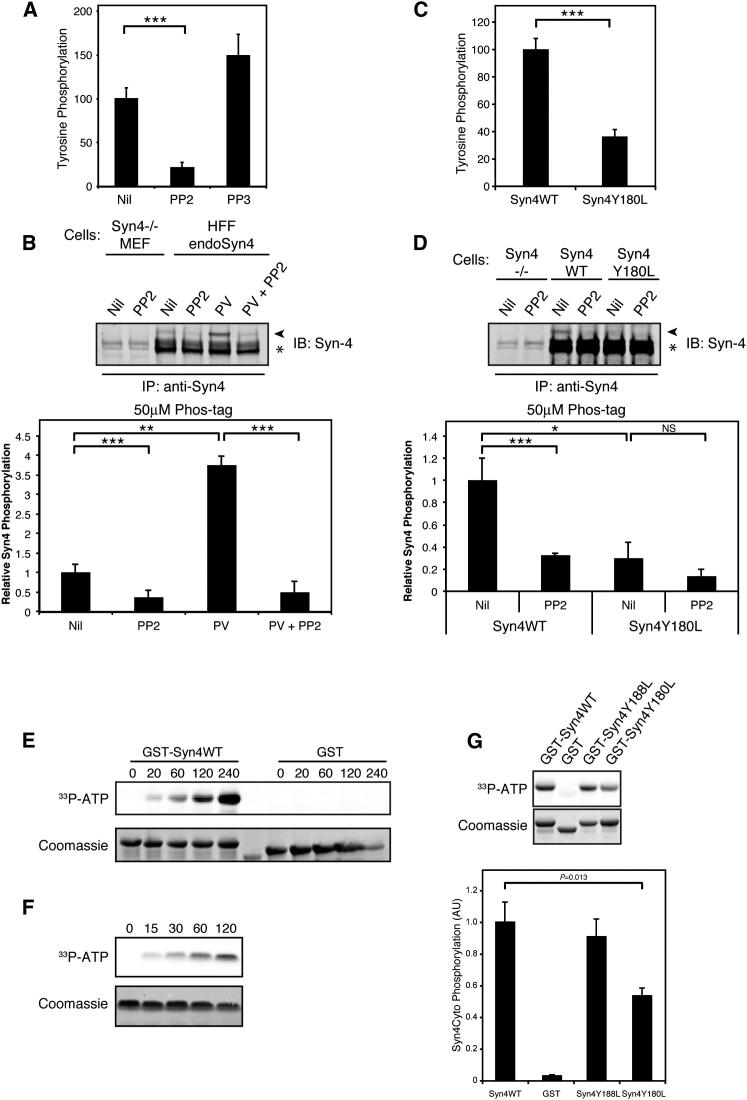
c-Src Directly Phosphorylates Syndecan-4 (A) Tyrosine phosphorylation of endogenous syndecan-4 isolated by solid-phase immunocapture from MEFs following treatment with PP2 or PP3 or with vehicle (DMSO) (Nil). (B) Phosphorylation of endogenous syndecan-4 assessed by Phos-tag immunoblotting (IB), following immunoprecipitation (IP) from human fibroblasts treated with or without pervanadate (PV) or PP2 or untreated (Nil). Syndecan-4-null MEFs were used as a negative control. Syndecan-4 resolved as a monomer and a dimer (see Figure S1A); asterisk denotes dimeric syndecan-4, arrowhead denotes a slow-migrating phosphorylated syndecan-4 band. (C) Tyrosine phosphorylation of HA-Syn4WT and HA-Syn4Y180L in HEK293T cells and isolated by solid-phase immunocapture. (D) Phos-tag immunoblot of syndecan-4 isolated from pervanadate-treated Syn4WT and Syn4Y180L cells in the presence of PP2 or untreated (Nil). See also Figure S1C. (A–D) Graphs show mean syndecan-4 phosphorylation, normalized to total syndecan-4 signal (A and C) or dimeric syndecan-4 band intensity (B and D), from three independent experiments ± SEM (^∗^p < 0.05, ^∗∗^p < 0.01, ^∗∗∗^p < 0.001, NS, not significant; Student’s t test). (E and F) Time course of phosphorylation of GST-syndecan-4 cytoplasmic domain (GST-Syn4WT) or GST alone (GST) (E) or recombinant syndecan-4 cytoplasmic domain (F) by recombinant active c-Src assessed by [γ-^33^P]ATP incorporation Numbers represent time in min. (G) Direct phosphorylation of GST-syndecan-4 cytoplasmic domain constructs (GST-Syn4WT, GST-Syn4Y188L, and GST-Syn4Y180L) by recombinant active c-Src assessed by [γ^33^P]ATP incorporation. Phosphorylation is expressed relative to total protein (Coomassie staining). Graph shows normalized means from 12 independent experiments ± SEM (p values were calculated using Student’s t test).

Phosphorylation is fundamental to the regulation of adhesive function and cell migration (Geiger et al., 2001). It has been reported that syndecan-4 is tyrosine phosphorylated and that this modification is sensitive to treatment with broad-spectrum tyrosine kinase inhibitors (Ott and Rapraeger, 1998). To understand the role syndecan-4 plays in integrating extracellular and intracellular signals during cell migration, and how this function is coordinated, we set out to identify the tyrosine kinase responsible for syndecan-4 phosphorylation. The syndecan-4 cytoplasmic domain contains three tyrosine residues that are potential phosphorylation targets. Analysis of the syndecan-4 cytoplasmic domain using NetPhorest, an online atlas of consensus sequence motifs (Miller et al., 2008), suggested that Src-family kinases (SFKs) might phosphorylate syndecan-4 on residue tyrosine 180 (Syn4Y180) (posterior probability = 0.085). This prompted us to test whether c-Src, a nonreceptor tyrosine kinase fundamentally associated with adhesion signaling, migration, and neoplastic progression (Playford and Schaller, 2004; Sandilands and Frame, 2008), regulates syndecan-4 phosphorylation. Using solid-phase capture, we assessed tyrosine phosphorylation of endogenous syndecan-4 isolated from mouse embryonic fibroblasts (MEFs). Treatment with the SFK inhibitor PP2 blocked syndecan-4 phosphorylation (Figure 1A), suggesting that SFKs regulate phosphorylation of syndecan-4. Syndecan-4 immunoprecipitation followed by Phos-tag gel electrophoresis allowed band-shift resolution and quantitation of endogenous syndecan-4 phosphorylation in human fibroblasts. Phos-tag induced the appearance of a single slow-migrating phosphorylated band, possibly suggesting that, under basal conditions, syndecan-4 may be phosphorylated on a single residue. Syndecan-4 resolved as a monomer and a dimer by immunoblotting, consistent with the reported formation of SDS-resistant syndecan dimers (Asundi and Carey, 1995). In untreated cells, only 2.6% of dimeric syndecan-4 was phosphorylated, and this was reduced by SFK inhibition. Treatment with the tyrosine phosphatase inhibitor pervanadate enhanced PP2-sensitive syndecan-4 phosphorylation to 11.7% (Figure 1B; Figure S1A available online).

To test whether Syn4Y180 is phosphorylated, HA-tagged wild-type syndecan-4 (HA-Syn4WT) or syndecan-4 with a mutated Y180 residue (HA-Syn4Y180L) was expressed in 293T cells and levels of tyrosine phosphorylation were assessed. Disruption of the Syn4Y180 residue substantially reduced syndecan-4 tyrosine phosphorylation, as determined by both solid-phase immobilization and immunoprecipitation (Figure 1C; Figure S1B). Syndecan-4 phosphorylation was then assessed in MEFs expressing either Syn4WT or Syn4Y180L, at endogenous levels, by Phos-tag gel electrophoresis. Treatment with PP2 inhibited Syn4WT phosphorylation by 68%, and phosphorylation of Syn4Y180 was reduced by 70% relative to Syn4WT (Figure 1D; Figure S1C). Therefore, syndecan-4 can be tyrosine phosphorylated by SFKs, and Syn4Y180, the SFK target predicted by NetPhorest, is the major site for this modification.

To test whether c-Src phosphorylates syndecan-4 directly, we assessed c-Src-mediated phosphorylation of isolated syndecan-4 cytoplasmic domains. Recombinant active c-Src phosphorylated GST-Syn4WT, but not GST alone, in a time-dependent manner (Figure 1E). Similarly, recombinant, custom-synthesized syndecan-4 cytoplasmic domain was phosphorylated by active c-Src (Figure 1F), and analysis of this peptide by liquid chromatography-tandem mass spectrometry identified Syn4Y180 as a target of c-Src-mediated phosphorylation (Figure S1D). Consistent with Syn4Y180 being a target for c-Src, phosphorylation of GST-Syn4Y180L by active c-Src was substantially reduced relative to GST-Syn4WT (or the control tyrosine mutant GST-Syn4Y188L) (Figure 1G). Interestingly, Y197 in the PDZ-binding domain of syndecan-4 could also be phosphorylated by recombinant active c-Src (Figure S1E), which likely accounts for the residual tyrosine phosphorylation detected upon disruption of Y180 (Figure 1G). However, Phos-tag gel electrophoresis (Figure 1D; Figure S1C) showed that in the cellular environment, Syn4Y180 is the major site of SFK-mediated syndecan-4 phosphorylation.

### Syndecan-4 Regulates Cell Migration and FA Dynamics

**Figure 2:**
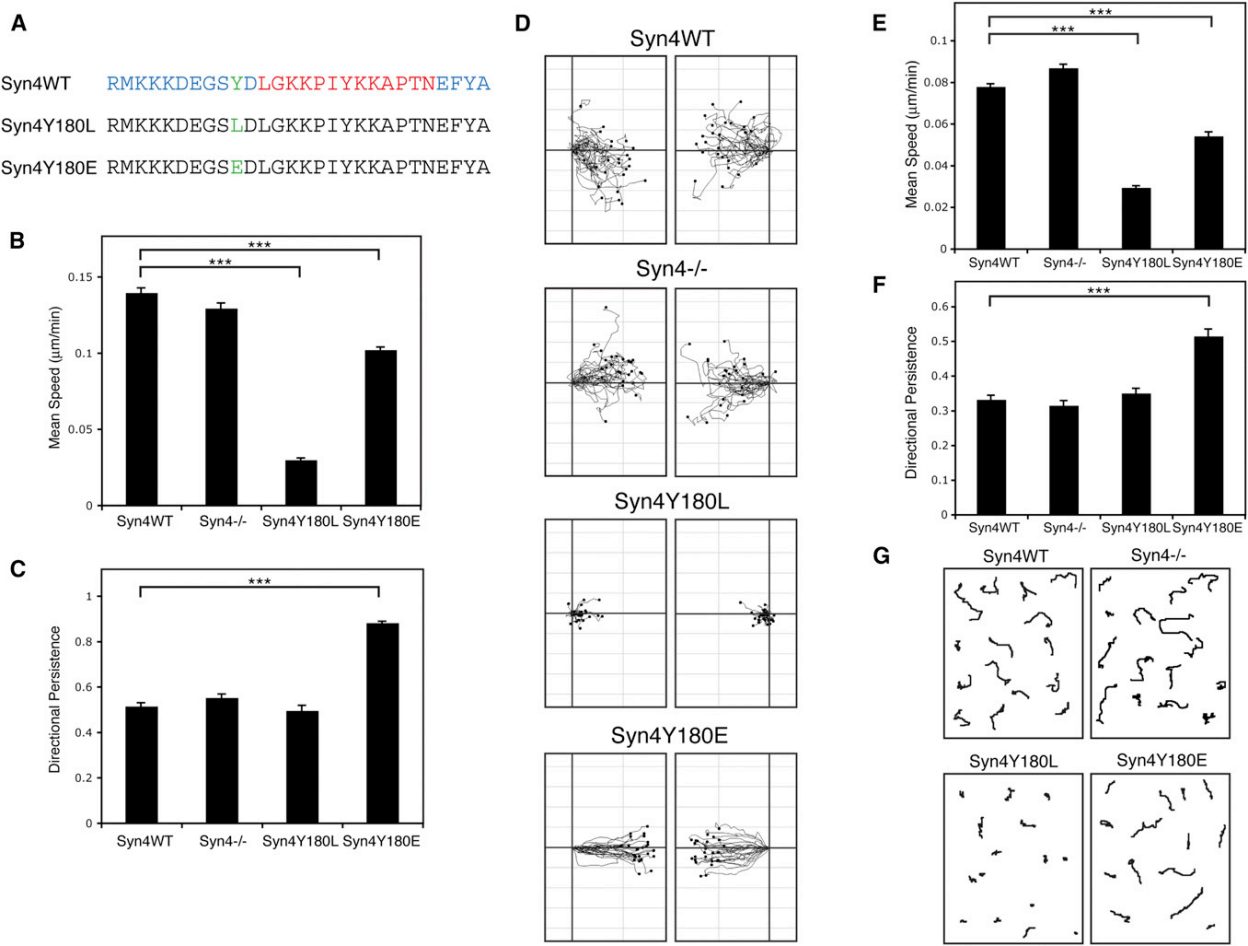
Syndecan-4 Y180 Dictates Migratory Behavior (A) Sequence alignment of syndecan-4 cytoplasmic domains (Syn4WT, Syn4Y180L, Syn4Y180E). Blue, conserved region; red, variable domain; green mutated Y180 residue. (B–D) Migration of syndecan-4^−/−^ MEFs, retrovirally transduced with Syn4WT, Syn4Y180L, Syn4Y180E, or empty vector (Syn4^−/−^), in scratch wound assays. Individual cells were tracked over 14 hr. Directional persistence, migration speed, and representative migration tracks are shown. (E–G) Migration of Syn4WT, Syn4^−/−^, Syn4Y180L, and Syn4Y180E cells plated on 2D fibronectin coated from solution was analyzed over 15 hr, 20 min by time-lapse microscopy. Directional persistence, migration speed, and representative migration tracks are shown. Values are means ± SEM; n > 100 cells (^∗∗∗^p < 0.001; Z test). See also Movie S1.

To investigate the contribution of c-Src-mediated syndecan-4 phosphorylation and signaling to cell migration, we expressed wild-type syndecan-4 or cytoplasmic domain mutants in syndecan-4-null fibroblasts. We generated cells expressing Syn4Y180L, which could not be phosphorylated on Y180, and Syn4Y180E, in an attempt to generate a phosphomimetic syndecan-4 that emulates c-Src-mediated phosphorylation of the Y180 residue (Figure 2A). Modulation of Syn4Y180 in this way induced striking changes in migratory behavior: on two-dimensional substrates, both in scratch wound assays (Movie S1; Figures 2B–2D) and when plated sparsely on fibronectin (Figures 2E–2G), expression of the nonphosphorylatable syndecan-4 mutant Syn4Y180L almost completely blocked net cell migration. By contrast, expression of the phosphomimetic mutant Syn4Y180E induced a directionally persistent mode of migration. Consistent with other studies (Caswell et al., 2008; Danen et al., 2005), this increase in persistence was accompanied by a slight reduction in migration speed.

**Figure 3:**
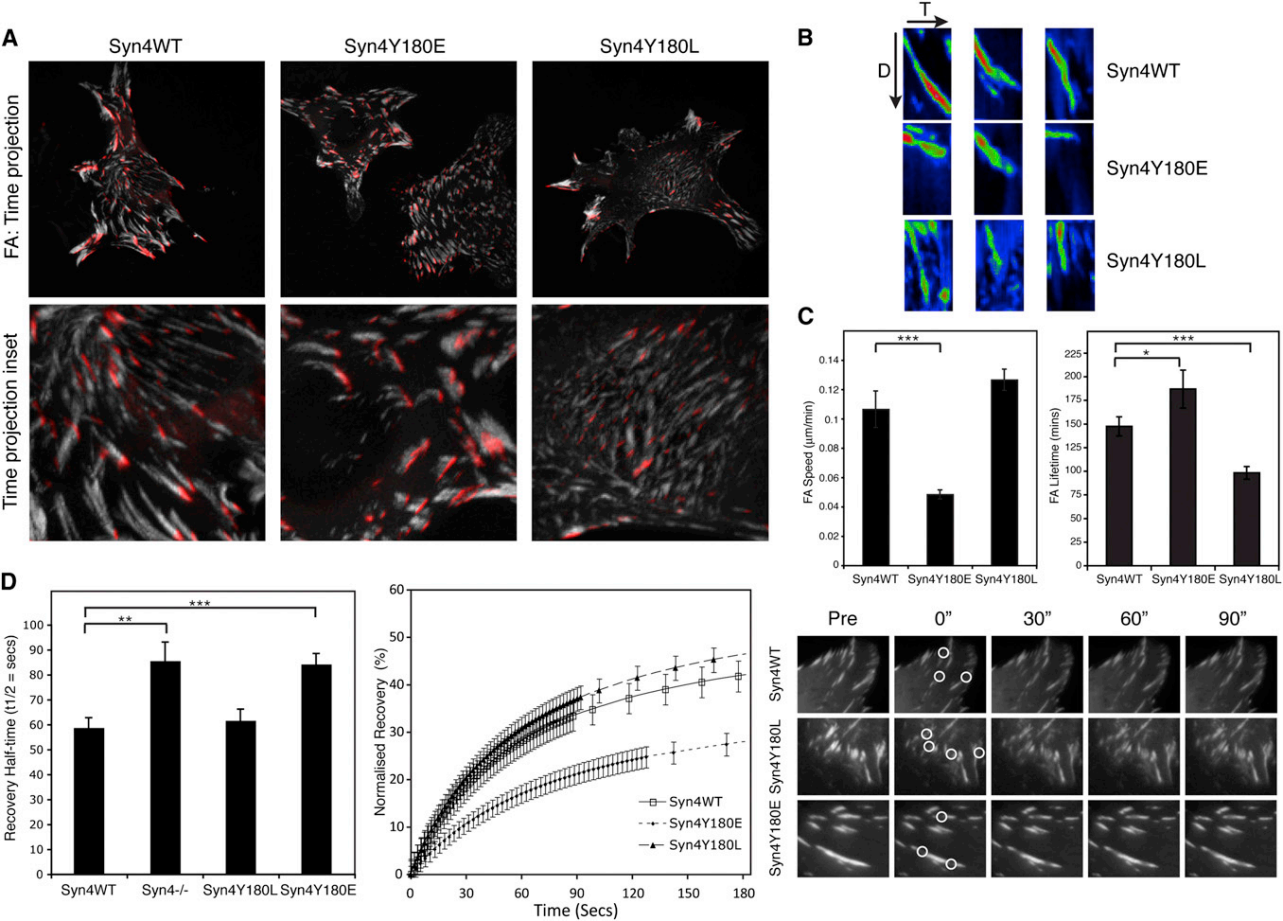
Phosphorylation Competence of Syndecan-4 Y180 Regulates FA Dynamics (A–C) Syn4WT, Syn4Y180L, and Syn4Y180E were transfected with GFP-vinculin, and FA translocation was monitored by fluorescence live-cell imaging. (A) Time projections of FA translocation of representative cells. Projection images show FA movement over 18 frames (127.5 min), with the FA positions in the first frame shown in red. Lower panel shows higher-magnification inset images of time projections. (B) Kymograph analysis of representative FAs in Syn4WT, Syn4Y180E, and Syn4Y180L cells with a spectral fluorescence intensity scale applied. Time (T), 217.5 min (30 frames); distance (D), 12 μm. (C) Quantitation of FA speed and lifetime in Syn4WT, Syn4Y180E, and Syn4Y180L cells expressing GFP-vinculin. Values are means ± SEM; n > 25 (^∗^p < 0.05, ^∗∗∗^p < 0.001; Student’s t test). (D) FRAP analysis of GFP-vinculin-transfected Syn4WT, Syn4^−/−^, Syn4Y180L, and Syn4Y180E cells. Mean GFP-vinculin recovery half-time and normalized recovery curves are shown (error bars show SEM; n = 31–67 FAs per condition; ^∗∗^p = 0.0013, ^∗∗∗^p < 0.001; Student’s t test). Data are representative of at least three independent experiments. Images show GFP-vinculin recovery in representative FAs. White circles highlight sites of photobleaching. See also Figure S2 and Movie S2.

Cell movement has an essential reliance on precise coordination of dynamic cell-ECM interactions. Overall, FA dynamics are a function of adhesion contact formation, maturation, stabilization, translocation, dissociation, and the diffusion and mobility of individual FA components. In view of the effects of Syn4Y180 phosphorylation on migration, we sought to determine whether modification of this residue was involved in the regulation of FA dynamics. As the FA adaptor protein vinculin provides a physical link between ECM-bound integrins and the actin cytoskeleton, we initially used live-cell imaging of GFP-vinculin to monitor FA dynamics in Syn4WT-, Syn4Y180L-, and Syn4Y180E-expressing cells (Movie S2; Figures 3A–3C; Figures S2A and S2B). The FAs of Syn4WT-expressing cells translocated centripetally at 0.106 ± 0.012 μm/min. By contrast, Syn4Y180E cells maintained relatively static FAs, with substantially reduced rates of FA translocation (0.048 ± 0.003 μm/min) and decreased turnover (as indicated by increased FA lifetime). FAs in cells expressing Syn4Y180L were smaller, translocated more rapidly (0.126 ± 0.007 μm/min), and displayed significantly increased turnover. Analysis of endogenous FA translocation, by interference reflection microscopy, also showed that expression of Syn4Y180E increased FA stability (Figure S2C).

To assess the dynamics of FA components, we next used fluorescence recovery after photobleaching (FRAP). Expression of mutant syndecan-4 constructs had no significant effect on the GFP-vinculin mobile fraction (Figure S2D), but significantly altered the half-time of recovery (Figure 3D). Expression of phosphomimetic Syn4Y180E substantially increased the half-time of recovery of vinculin (84.8 ± 4.3 s), demonstrating adhesion complex stabilization. By contrast, expression of phosphorylation-resistant Syn4Y180L did not alter the rate of GFP-vinculin recovery compared to the wild-type receptor (62.2 ± 4.5 s and 59.4 ± 4.0 s, respectively). Given the fundamental contribution of adhesion dynamics to the regulation of cell migration, it is perhaps not surprising that, on fibrillar cell-derived matrices, expression of phosphomimetic Syn4Y180E, which suppressed FA dynamics, substantially reduced migration speed and relative displacement by inducing a striking tail retraction defect (Figures S2E–S2G).

These data suggest that the phosphorylation status of Syn4Y180 dictates both the migratory characteristics of cells and the dynamics of their FAs. Indeed, Syn4Y180 appears to function as a switch between two distinct modes of behavior characterized either by accelerated turnover of FAs and a complete inability to migrate (Syn4Y180L) or by increased FA stabilization resulting in directionally persistent migration (Syn4Y180E).

### Src-Mediated Phosphorylation of Syndecan-4 Regulates FA Dynamics

**Figure 4:**
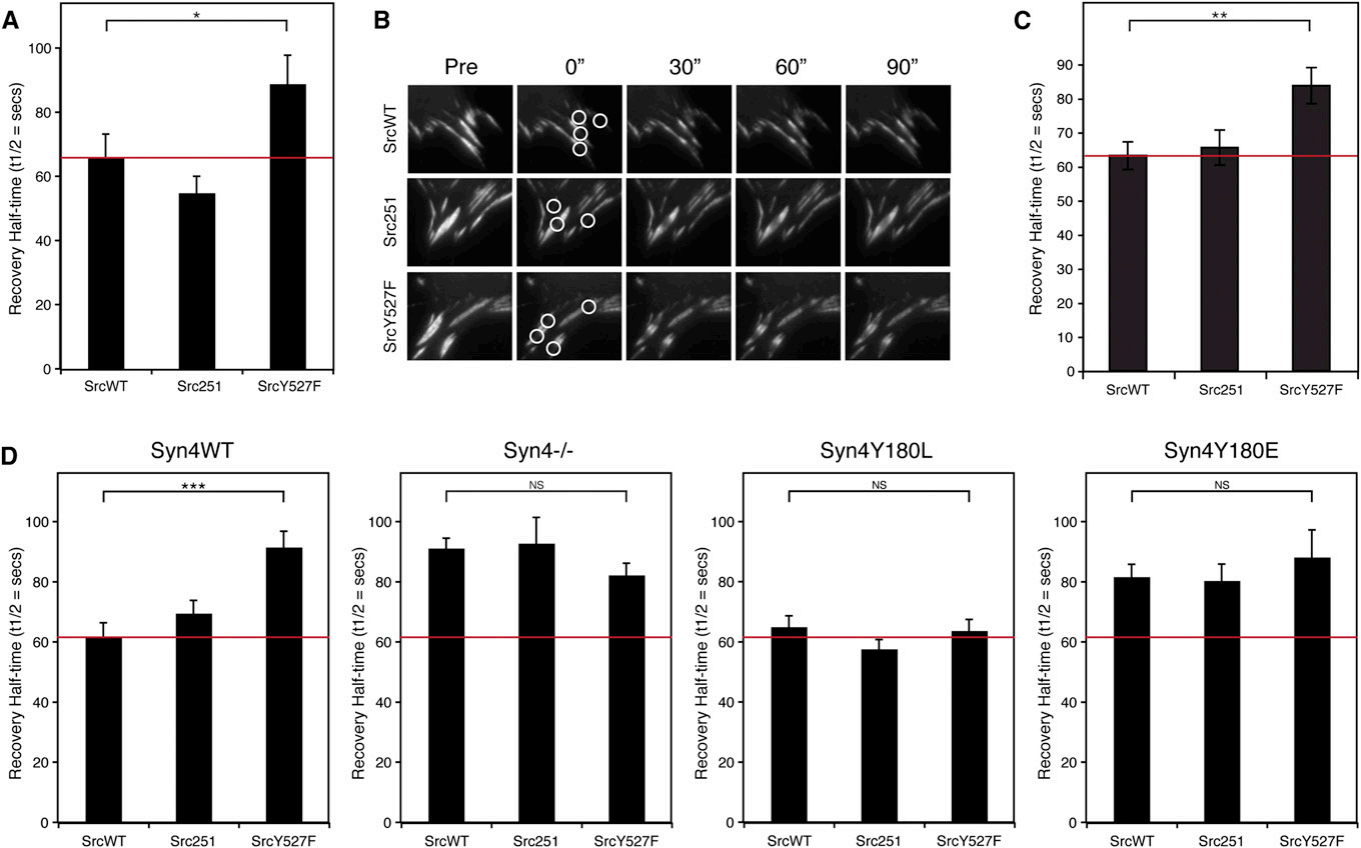
c-Src-Mediated Phosphorylation of Syndecan-4 Stabilizes FAs (A–C) FRAP analysis of GFP-vinculin in NIH 3T3 cells (A and B) or wild-type MEFs (C) cotransfected with c-Src constructs (wild-type SrcWT, constitutively primed SrcY527F, or kinase-deleted Src251). Mean GFP-vinculin recovery half-time is shown ± SEM; (A) n = 33–38 and (C) n = 36–47 FAs per condition. (B) Images showing GFP-vinculin recovery in representative FAs in NIH 3T3 cells. White circles represent sites of photobleaching. (D) FRAP analysis of Syn4WT-, Syn4^−/−^-, Syn4Y180L-, and Syn4Y180E-transduced cells cotransfected with GFP-vinculin and either SrcWT, Src251, or SrcY527F. Mean GFP-vinculin recovery half-time is shown ± SEM (n = 26–46 FAs per condition). Red lines represent rate of vinculin recovery in wild-type cells (NIH 3T3 cells, wild-type MEFs, or Syn4WT cells) expressing SrcWT determined within the same experiment. ^∗^p < 0.05, ^∗∗^p < 0.01, ^∗∗∗^p < 0.001, NS, not significant, using Student’s t test. See also Figure S2H.

To test the role of c-Src in regulating FA dynamics, constitutively active SrcY527F was coexpressed with GFP-vinculin in cells expressing endogenous syndecan-4. SrcY527F expression significantly increased the half-time of recovery of GFP-vinculin (Figures 4A–4C), consistent with FA stabilization (NIH 3T3 SrcWT: 66.2 ± 7.1 s; SrcY527F: 89.0 ± 8.9 s). This finding was also consistent with the increased half-time of recovery of GFP-vinculin induced by phosphomimetic syndecan-4 Y180E expression (Figure 3D). By contrast, kinase-dead Src251 did not significantly affect the dynamics of GFP-vinculin recovery. Constitutively active SrcY527F also decreased the rate of GFP-vinculin recovery in cells expressing Syn4WT (SrcY527F: 91.7 ± 5.3 s versus SrcWT: 62.1 ± 4.4 s). Importantly, however, introduction of active SrcY527F or kinase-dead Src251 had no effect on vinculin recovery in Syn4^−/−^, Syn4Y180L, or Syn4Y180E cells, which are all unable to be modified on Y180 by c-Src (Figure 4D; Figure S2H). Thus, c-Src-mediated suppression of FA dynamics is dependent on both syndecan-4 expression and the integrity of the Y180 residue. These data demonstrate that c-Src-dependent Syn4Y180 phosphorylation increases FA stability and are consistent with phosphomimetic Syn4Y180E suppressing FA turnover, FA translocation, and vinculin dynamics (Figure 3).

### Syndecan-4 Phosphorylation Regulates Integrin Recycling

**Figure 5:**
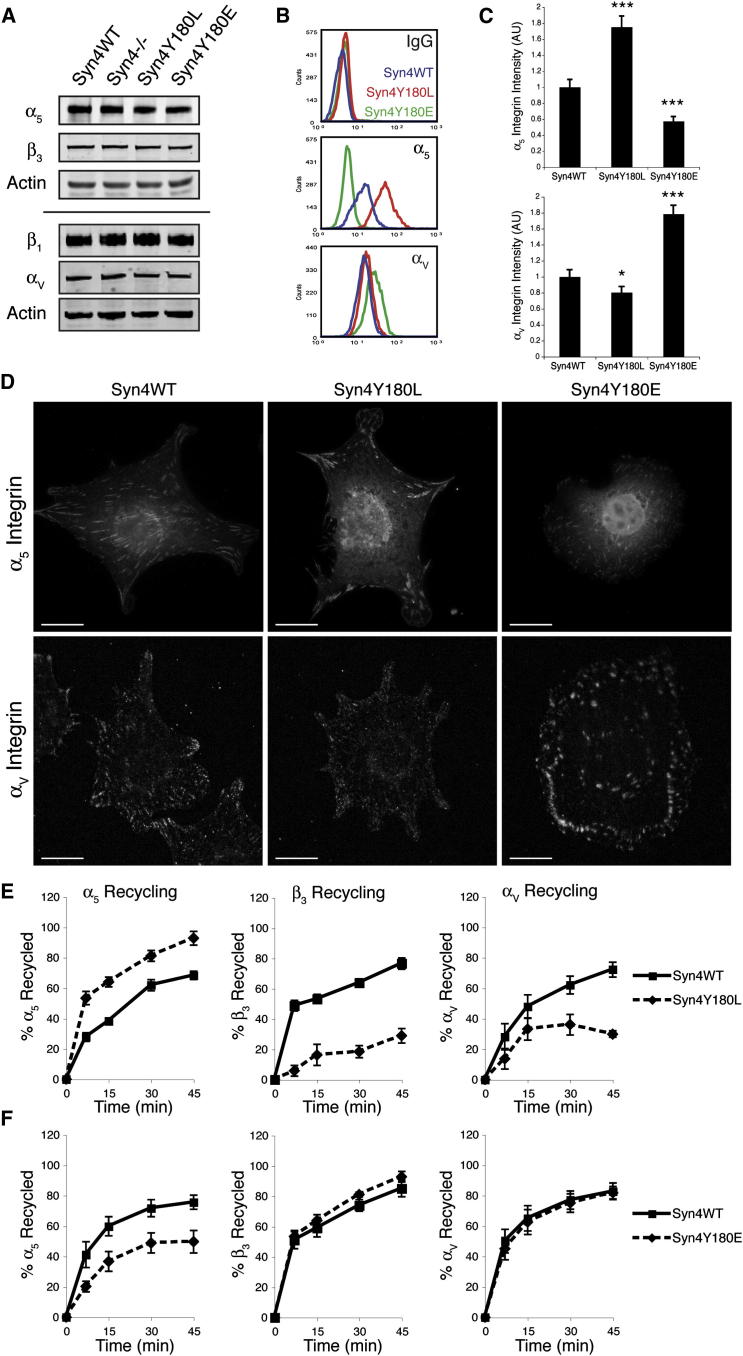
Syndecan-4 Regulates Heterodimer-Specific Integrin Recycling (A) Integrin α_5_, β_1_, α_V_, and β_3_ subunit expression detected by immunoblotting Syn4WT, Syn4^−/−^, Syn4Y180L, and Syn4Y180E total cell lysates. Immunodetection of actin was used as a loading control. (B) Flow cytometric analysis of cell-surface α_5_ and α_V_ integrin expression levels in cells expressing Syn4WT (blue), Syn4Y180L (red), and Syn4Y180E (green). (C) Levels of FA-localized α_5_ and α_V_ integrins in MEFs expressing Syn4WT, Syn4Y180L, and Syn4Y180E plated on fibronectin. Quantitative fluorescence analysis was used to calculate α_5_ and α_V_ fluorescence intensity (integrated density) in FAs. Data show mean fluorescence intensity normalized to Syn4WT cells ± SEM (n = 31–67 cells per condition; ^∗^p < 0.05, ^∗∗∗^p < 0.001; Student’s t test). Data are representative of three independent experiments. (D) Immunofluorescence micrographs showing subcellular localization of α_5_ and α_V_ integrin subunits in Syn4WT, Syn4Y180L, and Syn4Y180E MEFs on fibronectin (quantified in C). Scale bars represent 20 μm. (E) Recycling of α_5_, β_3_, and α_V_ integrin subunits assessed in cells expressing Syn4WT or Syn4Y180L. Data are means ± SEM of three independent experiments. (F) Integrin recycling in cells expressing Syn4WT or phosphomimetic Syn4Y180E. Data are means ± SEM of three independent experiments. See also Figure S3.

We next sought to determine the mechanism by which c-Src-mediated phosphorylation of syndecan-4 regulates FA dynamics. It has previously been shown that differential expression of specific integrin heterodimers regulates FA dynamics and cell migration (Danen et al., 2005; Worth et al., 2010). In epithelial cells, expression of α_5_β_1_ integrin promotes FA turnover and random cell motility, whereas expression of α_V_β_3_ suppresses FA dynamics and promotes directionally persistent migration (Danen et al., 2005). Because these cellular characteristics are remarkably similar to those seen in cells expressing Syn4WT and Syn4Y180E, respectively (Figures 2 and 3), we investigated whether disruption of the syndecan-4 Y180 residue altered expression of the prototypic fibronectin receptors α_5_β_1_ and α_V_β_3_. Syn4WT, Syn4^−/−^, Syn4Y180L, and Syn4Y180E cell lines each expressed similar total levels of α_5_, β_1_, α_V_, and β_3_ integrin protein, as assessed by western blotting (Figure 5A; Figure S3A); however, flow cytometry and quantitative immunofluorescence imaging demonstrated highly elevated levels of cell-surface-localized and FA-localized α_5_β_1_ in Syn4Y180L. By contrast, expression of Syn4Y180E reduced the levels of α_5_β_1_ and increased the levels of the α_V_ integrin subunit at the cell surface and in FAs (Figures 5C and 5D; Figure S3C).

The change in surface localization, rather than protein expression of α_5_ and α_V_ integrins, led us to investigate the intracellular trafficking of surface-labeled integrins. Modulation of the Syn4Y180 residue did not influence integrin internalization in a manner that could be responsible for heterodimer-specific integrin localization (Figures S3D and S3E). However, compared to wild-type syndecan-4, expression of Syn4Y180L substantially increased recycling of α_5_β_1_ to the membrane and, reciprocally, suppressed redelivery of α_V_β_3_ (Figure 5E). Conversely, recycling of α_5_β_1_ was inhibited by expression of phosphomimetic Syn4Y180E, whereas membrane trafficking of α_V_β_3_ was unaffected (Figure 5F). Thus, the phosphorylation status of Syn4Y180 dictates specifically which integrin heterodimers are recycled to the membrane.

Interestingly, recycling of α_5_β_1_ and α_V_β_3_ was not disrupted in syndecan-4-deficient cells (Figure S3F), suggesting that, in the absence of syndecan-4, cells undergo a degree of reprogramming to allow syndecan-independent recycling of α_5_β_1_ and α_V_β_3_. This may explain why migration of syndecan-4-null cells, on 2D substrates, was similar to that of cells expressing wild-type syndecan-4 (Figure 2). The fact that cells adopt alternative mechanisms to regulate integrin recycling upon deletion of syndecan-4 may be a testament to the importance of this process in normal physiological function.

### Syndecan-4 Phosphorylation Regulates Arf6 Activity

**Figure 6:**
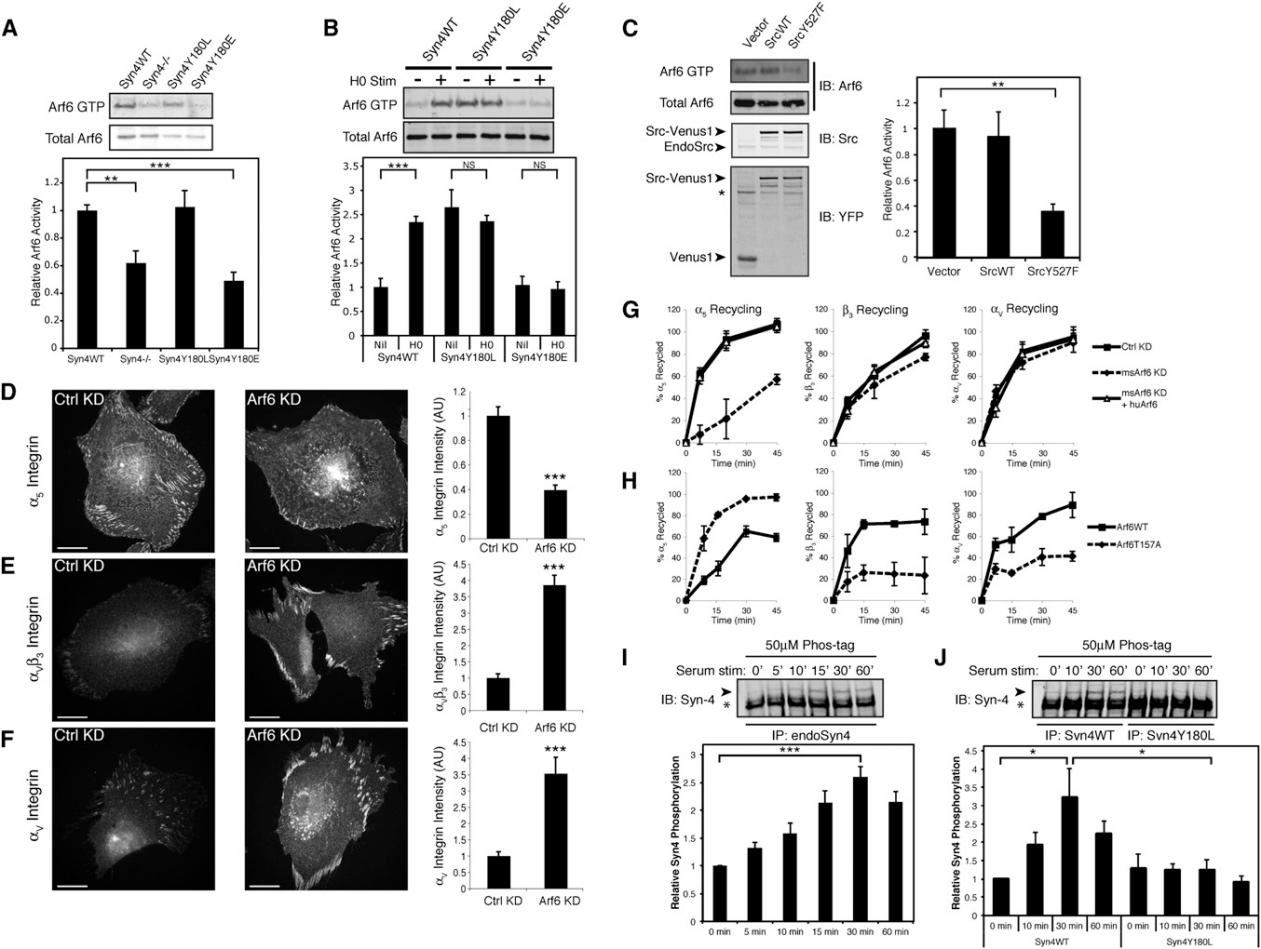
Syndecan-4 and c-Src Regulate Arf6 Activity to Drive Heterodimer-Specific Integrin Trafficking (A–C) Arf6 activity (Arf6 GTP) assessed by effector pull-down assay. (A) Steady-state Arf6 activity in Syn4WT-, Syn4^−/−^-, Syn4Y180L-, and Syn4Y180E-expressing MEFs. (B) H0-stimulated Arf6 activity in Syn4WT, Syn4Y180L, and Syn4Y180E cells plated on 50K. (C) Steady-state Arf6 activity in Venus1- (Vector), SrcWT-Venus1-, and SrcY527F-Venus1-expressing NIH 3T3 fibroblasts. Expression was determined by immunoblotting Src and YFP. Src-Venus1 arrowhead, Src-Venus1 bands; EndoSrc arrowhead, endogenous Src; Venus1 arrowhead, Venus1 construct (from empty vector). Asterisk denotes bands from previous endogenous Src immunoblotting. Graphs show mean Arf6 activity, normalized to total Arf6 ± SEM; (A) n = 4, (B) n = 3, (C) n = 3. ^∗∗^p < 0.01, ^∗∗∗^p < 0.001, NS, not significant. (D–F) Immunofluorescence analysis of FA-localized integrins in human fibroblasts on fibronectin following nontargeting (Ctrl KD) or Arf6 knockdown (Arf6 KD). Cells were stained for α_5_ (D), α_V_β_3_ (E), and α_V_ (F) and fluorescence intensity in FAs was calculated by quantitative fluorescence analysis. ^∗∗∗^p < 0.001; α_5_, n = 53–59; α_V_β_3_, n = 53–58; α_V_, n = 33–53. Data are means ± SEM representative of three independent experiments. Scale bars represent 30 μm. (G) Recycling of α_5_, β_3_, and α_V_ integrin subunits in NIH 3T3 cells transfected with nontargeting (Ctrl KD), mouse Arf6-targeting (msArf6 KD) siRNA, or mouse Arf6-targeting siRNA and HA-tagged human Arf6 cDNA (msArf6 KD + huArf6). Data are means ± SEM of three independent experiments. (H) Integrin recycling in NIH 3T3 cells transfected with Arf6WT or fast-cycling Arf6T157A (83% transfection efficiency). Data show means ± SEM representative of three independent experiments. (I and J) Phosphorylation of endogenous syndecan-4 (I) or Syn4WT and Syn4Y180L (J) assessed by Phos-tag immunoblotting, following immunoprecipitation from cells stimulated with 20% serum. Syndecan-4 resolved as a monomer and a dimer (see Figures S5K and S5L). Asterisks denote dimeric syndecan-4; arrowheads denote slow-migrating phosphorylated syndecan-4 bands. Graphs show mean syndecan-4 phosphorylation, normalized to dimeric syndecan-4 band intensity from three independent experiments ± SEM (^∗^p < 0.05, ^∗∗∗^p < 0.001). All p values were calculated with Student’s t test. See also Figures S4 and S5.

The small GTPase Arf6 is activated upon ECM engagement (Balasubramanian et al., 2007) and can regulate recycling of receptors from the perinuclear recycling compartment to the membrane (D’Souza-Schorey and Chavrier, 2006). Syndecans and integrins are among the receptors known to be trafficked by Arf6, but the molecular mechanisms and heterodimer specificity of this regulation have not been defined (Dunphy et al., 2006; Powelka et al., 2004; Zimmermann et al., 2005). The ability of syndecan-4 Y180 mutants to regulate differential recycling of α_5_β_1_ and α_V_β_3_ integrins prompted us to test whether syndecan-4, and specifically phosphorylation of the Y180 residue, regulates Arf6 activity. Effector pull-down assays showed that, compared to cells expressing Syn4WT, phosphomimetic Syn4Y180E-expressing cells had substantially reduced steady-state levels of Arf6 activity (Figure 6A), which could explain the reduced recycling of α_5_β_1_ integrin (Figure 5F). By contrast, cells expressing Syn4Y180L displayed high levels of Arf6 activity, similar to those in Syn4WT cells (Figure 6A).

Some of our data suggested that syndecan-4-null cells may be subject to compensation mechanisms as, despite having low levels of basal Arf6 Activity (Figure 6A) and reduced rates of vinculin recovery (Figure 3D), they displayed normal 2D migration (Figure 2) and integrin recycling (Figure S3E). Consequently, it was important to assess the effects of modulating the phosphorylation competence of syndecan-4 in cells of wild-type background. Therefore, we expressed human Syn4WT, Syn4Y180L, and Syn4Y180E in NIH 3T3-derived cells and used siRNA to transiently silence endogenous mouse syndecan-4 (Figure S4A). Importantly, FRAP analysis revealed that, although suppression of endogenous syndecan-4 slightly reduced the rate of vinculin recovery relative to cells expressing huSyn4WT, expression of phosphomimetic huSyn4Y180E substantially reduced vinculin recovery. Moreover, in this cellular context, expression of huSyn4Y180L increased the rate of vinculin recovery relative to expression of huSyn4WT (Figures S4B and S4C). Furthermore, expression of huSyn4Y180E suppressed steady-state Arf6 activity, whereas nonphosphorylatable huSyn4Y180L activated Arf6 activity upon suppression of endogenous msSyn4 expression (Figure S4D). Consistent with these effects on FA dynamics and Arf6 activity, expression of huSyn4Y180L reduced migration speed following suppression of endogenous msSyn4 expression, whereas huSyn4Y180E induced directionally persistent cell migration (Figures S4E and S4F). Notably, expression of huSyn4Y180L or Syn4Y180E did not affect Arf6 activity or cell migration when cells were transfected with nontargeting siRNA (Figures S4D–S4F). Thus, expression of syndecan-4 phosphorylation-site mutants differentially regulated Arf6 activity, FA dynamics, and cell migration in both syndecan-4-null cells and cells derived from a wild-type background following suppression of endogenous syndecan-4, further highlighting the importance of Syn4Y180 in controlling cellular function.

To assess directly the contribution of syndecan-4 engagement to Arf6 activity modulation, human fibroblasts (expressing endogenous syndecan-4) were prespread on a fragment of fibronectin containing the integrin-binding central cell-binding domain (50K/Fn6-10) and stimulated with a soluble fragment of fibronectin containing the syndecan-binding domain (H0), as described previously (Bass et al., 2007a). Stimulation with the syndecan ligand substantially increased Arf6 activity, but not following siRNA-mediated suppression of syndecan-4 expression, demonstrating that engagement of endogenous syndecan-4 is required and sufficient to stimulate Arf6 activation (Figure S4G). Importantly, although H0 stimulation of Syn4WT cells induced a substantial increase in Arf6 activity, it had no effect on the activation status of Arf6 in cells harboring mutations in the Y180 residue (Figure 6B). Cells expressing Syn4Y180L had constitutively elevated Arf6 activity, even in the absence of syndecan ligation, whereas phosphomimetic Syn4Y180E cells had suppressed levels of Arf6 activity, even after syndecan-4 engagement (Figure 6B), despite the fact that mutation of Syn4Y180 did not alter H0 binding (Figure S4H). Thus, Arf6 activation is dependent on syndecan-4 expression and engagement and the integrity of the Y180 residue. This suggests that syndecan-4 engagement dynamically regulates Arf6 activity and that this is controlled by the Y180 phosphorylation status.

As c-Src phosphorylates syndecan-4 on Y180, we tested whether c-Src activity could influence Arf6 activation. Steady-state Arf6 activity was assessed in cells expressing either wild-type or constitutively active c-Src (SrcWT-Venus1 and SrcY527F-Venus1, respectively). Consistent with the reduced levels of Arf6 activity observed in cells expressing phosphomimetic Syn4Y180E, expression of constitutively active c-Src significantly reduced Arf6 activity (Figure 6C; Figure S4I). Importantly, active c-Src did not suppress Arf6 activity in cells expressing Syn4Y180L (Figure S4I), suggesting that c-Src suppresses Arf6 activity by phosphorylating syndecan-4 on Y180. Moreover, c-Src-mediated inhibition of Arf6 activity was not limited to fibroblasts, as a similar response was observed in A2780 ovarian carcinoma cells (Figure S4J).

As c-Src-mediated syndecan-4 Y180 phosphorylation regulates both differential recycling of α_5_β_1_ and α_V_β_3_ integrins (Figures 5E and 5F) and Arf6 activity (Figures 6A–6C; Figures S4D, S4G, S4I, and S4J), we examined the role of Arf6 in regulating integrin trafficking. siRNA-mediated knockdown of Arf6 expression in human fibroblasts significantly reduced the levels of α_5_β_1_ in FAs (Figure 6D; Figure S5E) and induced a reciprocal increase of α_V_β_3_ in FAs (Figures 6E and 6F; Figures S5F and S5G). Moreover, suppression of Arf6 expression substantially decreased serum-stimulated recycling of α_5_β_1_, whereas it had no effect on the high levels of α_V_β_3_ trafficking (Figure S5H). Similarly, siRNA-mediated knockdown of mouse Arf6 in NIH 3T3 cells also suppressed α_5_β_1_ recycling, and this effect was rescued by expression of siRNA-resistant human Arf6 (Figure 6H; Figure S5A). Thus, inhibition of Arf6 expression recapitulated the integrin localization and recycling profile seen in cells expressing the phosphomimetic syndecan-4 construct Syn4Y180E, which suppresses Arf6 activation. Conversely, expression of Arf6T157A, a fast-cycling, constitutively active mutant that spontaneously exchanges nucleotides (Santy, 2002), substantially increased recycling of α_5_β_1_ and decreased recycling of α_V_β_3_, compared with cells expressing Arf6WT (Figure 6G). It is notable that the effect of expressing Arf6T157A on heterodimer-specific integrin trafficking is very similar to that seen with expression of Syn4Y180L (the syndecan-4 mutant that is resistant to c-Src-mediated phosphorylation and promotes Arf6 activity even in the absence of syndecan-4 engagement).

To test the generality of our findings, we tested whether Arf6 regulates heterodimer-specific integrin recycling in nonfibroblastic cells. Arf6 expression was suppressed in A2780 ovarian carcinoma cells and A375-SM malignant melanoma cells, which both use α_5_β_1_, α_V_β_3_, and syndecan-4 to engage fibronectin, and in both cell lines Arf6 inhibition blocked recycling of α_5_β_1_ but not α_V_β_3_ (Figures S5B, S5C, S5I, and S5J). Thus, the effect of Arf6 inhibition in these tumor cell lines was highly reminiscent of that in human fibroblasts and MEFs (Figure 6G; Figure S5H). These data, in conjunction with the fact that active c-Src suppressed Arf6 activity in nonfibroblastic cells (Figure S4J), suggest that c-Src-mediated syndecan phosphorylation suppressing Arf6 activity to control heterodimer-specific integrin recycling is a general mechanism for controlling differential receptor engagement during cell migration.

Under steady-state conditions, syndecan-4 exhibited a low stoichiometry of phosphorylation (Figures 1B and 1D; Figures S1A and S1C). However, the profound differences in integrin trafficking and cell migration elicited by either phospho-null or phosphomimetic Syn4Y180 mutations (Figures 2 and 5E) suggest that for integrins to be recycled appropriately, and for cells to migrate efficiently, phosphorylation of Y180 must be dynamically regulated. Therefore, we tested whether syndecan-4 phosphorylation was influenced by extracellular stimuli. In human fibroblasts, serum stimulation triggered a wave of endogenous syndecan-4 phosphorylation (Figure 6I; Figure S5K). Similarly, in HA-Syn4-expressing MEFs, serum stimulation induced an approximately 3-fold increase in phosphorylation of Syn4WT, whereas perturbation of the Syn4Y180 residue completely inhibited the ability of serum to trigger syndecan-4 phosphorylation (Figure 6J; Figure S5L). Thus, syndecan-4 Y180 phosphorylation is dynamically regulated by extracellular signals in a manner consistent with syndecan-4 operating as an environmental sensor to coordinate integrin recycling and engagement during cell migration.

### Syndecan-4-Mediated Arf6 Activity Regulates FA Dynamics

**Figure 7:**
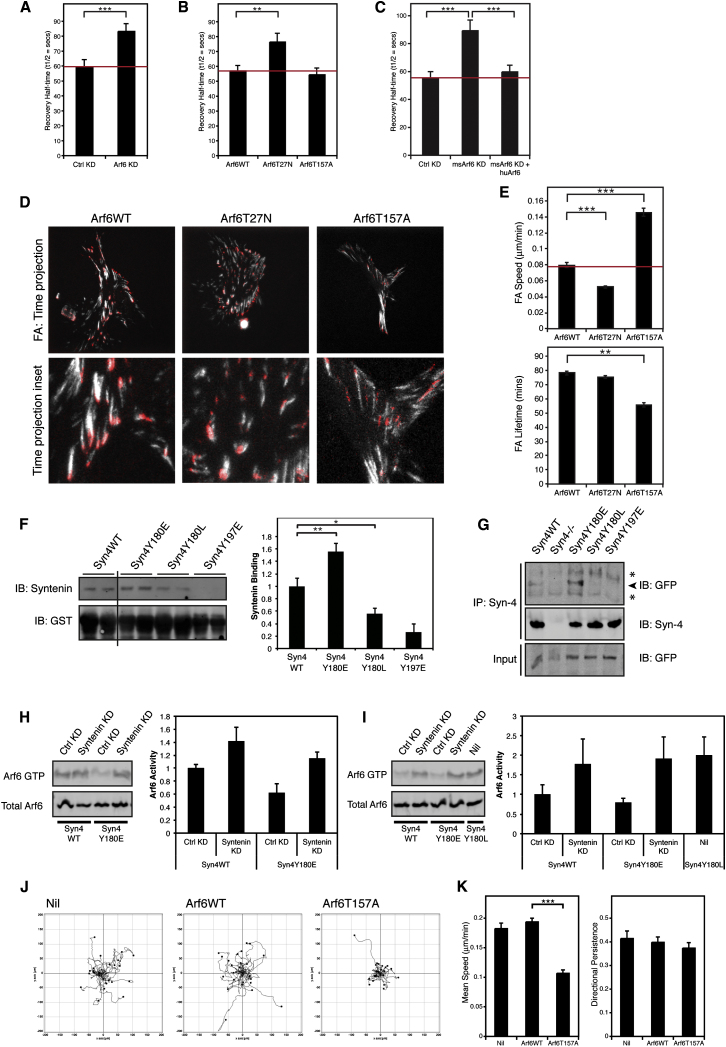
Syndecan-4-Mediated Arf6 Activity Regulates FA Turnover (A–C) FA component dynamics measured by GFP-vinculin FRAP in (A) Arf6 KD and Ctrl KD MEFs (n = 46–61), (B) wild-type MEFs cotransfected with Arf6 constructs (wild-type Arf6WT, dominant-negative Arf6T27N, or fast-cycling mutant Arf6T157A) (n = 48–60), or (C) NIH 3T3 cells transfected with control siRNA (Ctrl KD), mouse Arf6-targeting siRNA (msArf6 KD), or mouse Arf6-targeting siRNA and human Arf6 cDNA (msArf6 KD + huArf6) (n = 52–53). Mean GFP-vinculin recovery half-times are shown ± SEM. Red lines represents rate of vinculin recovery in cells expressing either Ctrl siRNA or Arf6WT. (D and E) FA translocation monitored by live-cell TIRF imaging in NIH 3T3 cells cotransfected with GFP-vinculin and Arf6WT, Arf6T27N, or Arf6T157A. (D) Time projections of FA translocation of representative cells. Projection images show FA movement over 16 frames (112.5 min) with the FA positions in the first frame shown in red. Lower panel: higher-magnification inset images of time projections. (E) Quantitation of FA speed and lifetime in cells expressing GFP-vinculin and either Arf6WT, Arf6T27N, or Arf6T157A. Values are means ± SEM. Red line represents FA speed in wild-type cells expressing only GFP-vinculin. (F) Binding of syntenin, from cell lysate, to recombinant GST-Syn4 cytoplasmic domains (Syn4WT, Syn4Y180E, Syn4Y180L, and Syn4Y197E). Vertical line denotes removal of lanes from blot. Data are means ± SEM from three independent experiments. (G) Coimmunoprecipitation of GFP-syntenin with syndecan-4 (Syn4WT, Syn4Y180E, Syn4Y180L, Syn4Y197E) from MEFs. (H and I) Arf6 activity (Arf6 GTP) in Syn4WT and Syn4Y180E MEFs expressing control (Ctrl KD) or syntenin siRNA (syntenin KD), assessed by effector pull-down assay under steady-state conditions (H) and on 50K (I). Syn4Y180L cells were used as a positive control in (I). Graphs show mean Arf6 activity, normalized to total Arf6, for three independent experiments ± SEM. (J and K) Migration of NIH 3T3 cells, cotransfected with GFP-vinculin and either Arf6WT, Arf6T157A, or no other cDNA (Nil), on 2D fibronectin analyzed over 16 hr by time-lapse microscopy. Representative migration tracks, migration speed, and directional persistence are shown. Values are means ± SEM (n = 36–84). ^∗^p < 0.05, ^∗∗^p < 0.01, ^∗∗∗^p < 0.001. All p values were calculated with Student’s t test. See also Figure S6 and Movie S3.

As disruption of the c-Src phosphorylation site within syndecan-4 induced defects in Arf6 activation, integrin trafficking, and FA dynamics (Figures 3, 5, and 6), and because Arf6 controlled heterodimer-specific recycling of integrins to the membrane (Figure 6), a process that is regulated by syndecan-4 Y180 (Figure 5), we used FRAP and live-cell imaging to test whether Arf6 regulates FA dynamics. In fibroblasts expressing endogenous syndecan-4, both siRNA-mediated Arf6 knockdown and expression of a dominant-negative Arf6T27N mutant (D’Souza-Schorey et al., 1995) stabilized FAs, increasing the half-time of recovery of GFP-vinculin (Figures 7A and 7B; Figures S6A and S6B). Moreover, knockdown of mouse Arf6 in NIH 3T3 cells also decreased GFP-vinculin turnover, and this effect was rescued by expression of siRNA-resistant human Arf6 (Figure 7C; Figure S6C). We further probed the role of Arf6 in regulating FA dynamics by performing live-cell total internal reflection fluorescence (TIRF) imaging of GFP-vinculin in cells expressing Arf6WT, dominant-negative Arf6T27N, or fast-cycling Arf6T157A (Movie S3; Figures 7D and 7E; Figures S6D and S6E). Compared to Arf6WT-expressing cells, Arf6T27N cells exhibited relatively static FAs with substantially reduced rates of FA translocation (Arf6WT: 0.079 ± 0.003 μm/min; Arf6T27N: 0.052 ± 0.002 μm/min). By contrast, FAs in cells expressing Arf6T157A translocated more rapidly (0.145 ± 0.005 μm/min) and dissociated more quickly (Arf6WT FA lifetime: 78.2 ± 1.4 min; Arf6T157A FA lifetime: 55.6 ± 1.4 min). Together, these findings are consistent with the alterations in FA dynamics induced by modulating the phosphorylation status of syndecan-4 Y180 (Figure 3). We next introduced dominant-negative and fast-cycling Arf6 constructs into cells expressing Syn4WT, Syn4Y180L, or Syn4Y180E and assessed GFP-vinculin turnover by FRAP (Figures S6F and S6G). Similar to cells expressing endogenous syndecan-4, expression of dominant-negative Arf6T27N in Syn4WT cells substantially increased the half-time of recovery of GFP-vinculin (Arf6WT: 60.4 ± 5.4 s; Arf6T27N: 88.2 ± 5.0 s). Moreover, the same construct stabilized the FAs of Syn4Y180L cells, suggesting that Arf6 lies downstream of syndecan-4. In cells expressing Syn4Y180E, which already have stable FAs, introduction of Arf6T27N had no further effect on vinculin dynamics; however, overexpression of either the fast-cycling Arf6T157A mutant or Arf6WT was sufficient to increase the turnover of vinculin. Thus, modulation of Arf6 activity is sufficient to rescue the FA defects induced by perturbation of the c-Src-phosphorylation motif of syndecan-4. These data are consistent with Arf6 functioning downstream of c-Src-mediated syndecan-4 phosphorylation in order to regulate heterodimer-specific integrin recycling and FA dynamics.

### Syndecan-4 Y180 Modulates Syntenin Binding

Phosphorylation often modulates protein-protein interactions in order to spatially coordinate cellular functions. Syntenin is a syndecan-binding adaptor protein known to influence Arf6-dependent recycling of syndecans (Zimmermann et al., 2005). Therefore, we tested whether Syn4Y180 modulates syntenin binding. In GST pull-down assays (Figure 7F) and coimmunoprecipitation from cells expressing GFP-syntenin (Figure 7G), phosphomimetic Syn4Y180E enhanced syntenin binding, whereas phospho-null Syn4Y180L mutation inhibited syntenin binding, suggesting that phosphorylation of Syn4Y180 promotes the association with syntenin. In both assays, a phosphomimetic mutation in the PDZ-binding domain (Syn4Y197E) was used as a negative control, as phosphorylation of the equivalent residue in syndecan-1 has been shown to inhibit syntenin binding (Sulka et al., 2009). As the phosphorylation competence of Syn4Y180 modulates syntenin binding and Arf6 activity, and syntenin regulates Arf6-dependent receptor recycling, we tested whether syntenin binding directly influences Arf6 activity. Under steady-state conditions, compared to Syn4WT, expression of Syn4Y180E suppressed Arf6 activity (Figure 6A; Figure S4D); however, syntenin knockdown elevated Arf6 activity in cells expressing Syn4Y180E to levels similar to Syn4WT (Figure 7H). Moreover, in Syn4WT and Syn4Y180E cells plated on 50K, which both exhibit low levels of Arf6 activity (Figure 6B), suppression of syntenin expression promoted Arf6 activity to levels that were comparable to Syn4Y180L (Figure 7I). Because simulating Syn4Y180 phosphorylation enhances syntenin binding and suppresses Arf6 activity and, in this cellular context, inhibiting syntenin expression or syntenin-syndecan-4 binding elevates Arf6 activity, these data suggest that syndecan-4 Y180 phosphorylation promotes syntenin binding in order to suppress Arf6 activity.

Cell migration requires precise coordination of integrin engagement and adhesion contact dynamics. As syndecan-4 phosphorylation and syntenin binding regulate Arf6 activity to control integrin trafficking and FA dynamics, we assessed the effect of modulating Arf6 activity on cell migration. Expression of fast-cycling Arf6T157A (which decreases FA stability by promoting α_5_β_1_, and suppressing α_V_β_3_, recycling) substantially reduced migration of cells expressing endogenous syndecan-4, Syn4WT, and Syn4Y180E to levels similar to cells expressing nonphosphorylatable Syn4Y180L (Figures 7J and 7K, S6H and S6I, and 2B–2G, respectively). Importantly, expression of Arf6WT in Syn4Y180E cells, which enhanced FA dynamics (Figure S6F), was sufficient to induce a switch from directionally persistent to random migration (similar to Syn4WT-expressing cells) (Figures S6H and S6I). Overall, the data in this study demonstrate that c-Src-mediated phosphorylation of syndecan-4 differentially regulates Arf6-dependent recycling of α_5_β_1_ and α_V_β_3_ integrins to control FA turnover and that precise spatiotemporal coordination of these events is required to allow efficient cell migration.

## Discussion

In this study, we have identified syndecan-4 phosphorylation as a key control point driving membrane delivery of specific integrin heterodimers to control FA dynamics and coordinate cell migration. Specifically, we show the following:

(1) c-Src phosphorylates syndecan-4 directly on Y180, and this modification leads to a stabilization of FAs.
(2) ECM engagement of syndecan-4 activates the small GTPase Arf6, and c-Src-mediated syndecan-4 phosphorylation suppresses Arf6 activity.
(3) Syndecan-4-mediated Arf6 activation drives recycling of α_5_β_1_ integrin to the plasma membrane, and suppresses trafficking of α_V_β_3_, to promote FA turnover.
(4) Syndecan-4-dependent inactivation of Arf6 suppresses recycling of α_5_β_1_, increases levels of α_V_β_3_ at the cell-ECM interface, and stabilizes FAs.

Taking these findings together, c-Src-mediated phosphorylation of syndecan-4 functions as a molecular switch to regulate Arf6 activity differentially and dictate, in response to the extracellular environment, whether α_5_β_1_ or α_V_β_3_ integrins are recycled to the membrane to coordinate FA dynamics. During cell migration, FA turnover and stabilization need to be controlled precisely to orchestrate protrusion, tail retraction, and the application of locomotive cytoskeletal forces (Geiger et al., 2001; Ridley et al., 2003). Our data suggest that in migrating cells, c-Src-mediated phosphorylation of syndecan-4 is tightly and dynamically regulated in order to control heterodimer-specific recycling and engagement of α_5_β_1_ or α_V_β_3_ integrins in a spatially and temporally restricted manner, and that this allows precise coordination of FA dynamics to promote efficient migration.

It is becoming increasingly clear that intracellular trafficking plays a pivotal role in regulating integrin engagement and signaling during cell migration (Caswell et al., 2009; Pellinen et al., 2006) and that reciprocal antagonism between the recycling pathways of α_5_β_1_ and α_V_β_3_ regulates heterodimer-specific signaling and cell migration (Caswell et al., 2008, 2009; Morgan et al., 2009; White et al., 2007). Our data demonstrate that syndecan-4 coordinates these processes in an Arf6-dependent manner. As syndecan-4-dependent Arf6 activation was regulated by extracellular syndecan-4 engagement (Figure 6B; Figure S4G), this suggests that syndecan-4 functions as a “molecular antenna” sensing the microenvironment in order to spatially control the downstream localization, engagement, and function of anchoring molecules such as integrins. Integrin α_V_β_3_ is relatively immobile in FAs (Hu et al., 2007) and has a faster on/off binding rate for ligand than α_5_β_1_, allowing it to act as a mechanosensor requiring clustering and cytoskeletal adaptor recruitment to promote reinforcement and stabilization of adhesion, whereas α_5_β_1_ supports high ECM forces (Puklin-Faucher and Sheetz, 2009; Roca-Cusachs et al., 2009). So, by driving heterodimer-specific integrin recycling and clustering, syndecan-4 signaling has the capacity to coordinate the biomechanical response to the microenvironment during cell migration.

Although it is currently not clear which extracellular signals promote c-Src-mediated syndecan-4 phosphorylation in order to suppress Arf6 activity and modulate integrin trafficking, Src can associate with, and be activated or inactivated by, a range of different receptors, including integrins and growth factor receptors (Brunton et al., 2004; Playford and Schaller, 2004; Streuli and Akhtar, 2009). As syndecans act as receptors for ECM molecules and coreceptors for growth factors and cytokines (Alexopoulou et al., 2007; Bass et al., 2009; Morgan et al., 2007; Murakami et al., 2008), it is possible that they function as a nexus to integrate both ECM-associated and secreted microenvironmental signals. Intriguingly, c-Src binds to and can be activated by α_V_β_3_ integrin (Arias-Salgado et al., 2003; Morgan et al., 2009). Because syndecan-4 is phosphorylated by c-Src to drive α_V_β_3_ recycling, syndecan-4-dependent receptor trafficking and FA stabilization potentially could be regulated by positive reinforcement mechanisms. By contrast, Arf6 can regulate recycling of syndecans to the membrane (Zimmermann et al., 2005) and syndecan-4 engagement promotes Arf6 activity, suggesting another potential positive feedback mechanism. It follows that, in vivo, c-Src activity and syndecan-4 phosphorylation must be tightly regulated. Consistent with this notion, the basal stoichiometry of syndecan-4 Y180 phosphorylation was low yet dynamically regulated by extracellular stimuli, suggesting a model whereby syndecan-4 senses and interprets the extracellular environment to control Arf6 activity in a spatially and temporally restricted manner. It will now be instructive to determine the mechanisms by which extracellular signals are integrated to constrain syndecan-4 phosphorylation and coordinate integrin trafficking.

Phosphorylation of syndecan-4 Y180 promoted syntenin binding in order to suppress Arf6 activity. Although syndecans associate with syntenin via C-terminal PDZ-binding motifs, distal phosphorylation events have been previously shown to modulate syntenin binding (Koo et al., 2006). However, the mechanism by which the syndecan-syntenin association influences Arf6 activity is unclear; it may have a direct effect on the activity of Arf6 regulators (e.g., guanine nucleotide exchange factors or GTPase-activating proteins), or it may provide a platform for the spatial compartmentalization of intracellular signals. The syndecan-4 Y180 residue is conserved between all syndecans, as is the ability to bind syntenin, and thus it is tempting to speculate that, in different cellular or environmental contexts, phosphorylation of the equivalent residue in other syndecans may also modulate receptor trafficking, perhaps impacting different integrins or classes of receptor.

We have delineated a mechanism that defines syndecan-4 as a signaling nexus to integrate c-Src signaling and extracellular stimuli to control membrane delivery of integrins, regulate FA dynamics, and modulate cell migration. It is notable that many of the molecules and processes identified in this study, including SFKs, Arf6, and differential integrin expression and recycling, play a fundamental role in both wound healing and cancer invasion (Caswell et al., 2009; Muralidharan-Chari et al., 2009; Playford and Schaller, 2004). Thus, understanding syndecan-4-mediated coordination of these processes will improve our understanding of migration in both health and disease.

## Experimental Procedures

### Antibodies and Reagents

A list of antibodies and other reagents is presented in Supplemental Experimental Procedures.

### Cell Culture

Transfection, cell-line generation, and culture conditions are described in Supplemental Experimental Procedures.

### Solid-Phase Analysis of Tyrosine Phosphorylation

Biotinylated or unlabeled syndecan-4 from cell lysates was immobilized by solid-phase immunocapture using anti-mouse syndecan-4 or anti-HA antibodies. Phosphotyrosine or total syndecan-4 were immuno (or biotin) detected and developed with 2,2′-azino-bis(3-ethylbenzothiazoline-6-sulfonic acid) substrate (ABTS). Syndecan-4 tyrosine phosphorylation was calculated relative to total syndecan-4. Further details are available in Supplemental Experimental Procedures.

### Immunoprecipitation

Proteins were immunoprecipitated from cell lysate using 1–3 μg antibody and 45–80 μl protein G Sepharose beads for 1.5 hr at 4°C. Immune complex-bound beads were washed with lysis buffer and, where appropriate, heparitinase treated, and proteins were eluted with reducing sample buffer. Immunoprecipitated proteins and total cell lysates were resolved by SDS-PAGE or Phos-tag gel electrophoresis and detected by western blotting. Further details are available in Supplemental Experimental Procedures.

### Immunoblotting

Proteins were solubilized with SDS sample buffer, resolved by SDS-PAGE, and transferred to nitrocellulose. Proteins were detected using the Odyssey western blotting fluorescence detection system (LI-COR Biosciences), as described previously (Bass et al., 2008).

### Phosphorylation of Recombinant Syndecan-4 Cytoplasmic Domains

Purified GST-tagged or recombinant custom-synthesized syndecan-4 cytoplasmic domain peptides were incubated with 43 ng active recombinant Src, 50 μM ATP, and 5 μCi [γ-^33^P]ATP (excluded for mass spectrometry samples) at 30°C for the appropriate time. Reactions were stopped with 3% phosphoric acid, and proteins were analyzed by mass spectrometry or resolved by SDS-PAGE and analyzed on a phosphoimager. Further details are available in Supplemental Experimental Procedures.

### Two-Dimensional Cell Migration

We coated 24-well plates with plasma fibronectin from solution (10 μg/ml) and plated 5 × 10^3^ cells at 37°C and 5% CO_2_ in MEF medium for 4 hr prior to filming. For scratch wound assays, 5 × 10^4^ cells were plated on fibronectin-coated 24-well plates for 20 hr in MEF medium. Cell monolayers were wounded with sterile pipette tips. Time-lapse bright-field images were acquired on an AS-MDW live-cell imaging system (Leica) using a 5×/NA 0.15 Plan Fluotar objective and 1.5× magnification. Point visiting was used to allow multiple positions to be imaged within the same time course, and cells were maintained at 37°C and 5% CO_2_. Images were collected every 10 min over 14 hr (sparsely plated cells) or every 7.5 min over 15 hr and 20 min (scratch wound assays) using a Coolsnap HQ (Photometrics). Cell migration was tracked manually using the MTrackJ plug-in for ImageJ. Migration tracks were plotted using the Chemotaxis Tool and Manual Tracking ImageJ plug-ins.

### Fluorescence Live-Cell Imaging

Cells expressing GFP-vinculin were plated on fibronectin 24–48 hr posttransfection in the presence of 2% FBS. Time-lapse epifluorescence images were acquired on an Olympus inverted microscope (IX71) using DeltaVisionRT software (Applied Precision) (1 image every 7.5 min over 8 hr). Live-cell TIRF images were acquired on a TE2000 microscope (Nikon) using a 488 nm laser (1 image every 1.5 min over 2 hr) using point visiting and a Cascade 512B EM CCD camera (Photometrics). Details of FA translocation and lifetime analysis and data presentation are available in Supplemental Experimental Procedures.

### Fluorescence Recovery after Photobleaching

Cells expressing GFP-vinculin were plated on fibronectin 24–48 hr posttransfection and imaged at 37°C in Ham’s F12 medium supplemented with 2% FBS. FRAP was performed on an Olympus inverted microscope (IX71) equipped with a 488 nm FRAP laser under the control of DeltaVisionRT software (Applied Precision) using a 100×/NA 1.40 Plan Apo objective. Images were acquired every 3 s for 180 s postphotobleaching. One micrometer-diameter regions of interest were selected, and half-time of recovery (t_1/2_) and mobile fraction were calculated using softWoRx FRAP photokinetic analysis software (Applied Precision). The mobile fraction indicates the amount of a molecule that is freely mobile, whereas changes in the t_1/2_ signify alterations in diffusion or binding to, or release from, an immobile substrate within a complex (e.g., integrins).

### Flow Cytometry

Cells were detached using trypsin/EDTA or enzyme-free Hanks’-based cell-dissociation buffer (Invitrogen), washed, immunolabeled, and analyzed on a Beckman Coulter Cyan ADP. Further details are available in Supplemental Experimental Procedures.

### Immunofluorescence and Image Analysis

For standard immunofluorescence, cells were plated on fibronectin-coated coverslips in the presence of 2% FBS for 3 hr, fixed, and immunostained. Immunofluorescent images were acquired on an Olympus IX71 using DeltaVisionRT software and analyzed using ImageJ. Further details are available in Supplemental Experimental Procedures.

### Recycling Assays

Recycling of surface-biotinylated receptors from the perinuclear recycling compartment was assessed as previously described (Roberts et al., 2001) with the following modifications. All washes after the initial biotinylation step were performed with ice-cold Krebs buffer. Receptors were internalized in DMEM for 30 min at 37°C. Serum-stimulated recycling of internalized receptors back to the membrane was stimulated with DMEM and 15% FBS at 37°C. Integrins were captured for ELISA (antibodies are listed in Supplemental Experimental Procedures), and biotinylation was detected using ExtrAvidin-Peroxidase and ABTS (Bass et al., 2007a). Total levels of integrin internalization after 30 min at 37°C were also calculated.

### Arf6 Effector Pull-Down Assays

Arf6 activity was assessed by GST-GGA3 pull-down assay, as described previously (Santy and Casanova, 2001). Arf6 activity was determined for cells on fibronectin in the presence of 2% FBS for 4 hr (steady-state), or on 50K followed by 90 min stimulation with or without H0. Further details are available in Supplemental Experimental Procedures.

### Statistical Analyses

Data were analyzed using Student’s unpaired t tests or Z tests. p < 0.05 was considered statistically significant. p values and statistical tests are quoted in the figure legends.

## Supplemental Information

Document S1. Figures S1–S6 and Supplemental Experimental Procedures

Movie S1. Syndecan-4 Y180 Dictates Migratory Behavior, Related to Figure 2 Migration of Syn4WT-, Syn4Y180E-, and Syn4Y180L-expressing cells into scratch wounds (time = 16 hr).

Movie S2. Syndecan-4 Y180 Regulates FA Dynamics, Related to Figure 3 Dynamics of GFP-vinculin-containing FAs in Syn4WT-, Syn4Y180E-, and Syn4Y180L-expressing cells (time = 8 hr).

Movie S3. S3 Arf6 Activity Regulates FA Dynamics, Related to Figure 7 Dynamics of GFP-vinculin-containing FAs in Arf6WT-, Arf6T27N-, and Arf6T157A-expressing cells (time = 2 hr).

## References

[bib1] Alexopoulou A.N., Multhaupt H.A., Couchman J.R. (2007). Syndecans in wound healing, inflammation and vascular biology. Int. J. Biochem. Cell Biol..

[bib2] Arias-Salgado E.G., Lizano S., Sarkar S., Brugge J.S., Ginsberg M.H., Shattil S.J. (2003). Src kinase activation by direct interaction with the integrin β cytoplasmic domain. Proc. Natl. Acad. Sci. USA.

[bib3] Asundi V.K., Carey D.J. (1995). Self-association of *N*-syndecan (syndecan-3) core protein is mediated by a novel structural motif in the transmembrane domain and ectodomain flanking region. J. Biol. Chem..

[bib4] Balasubramanian N., Scott D.W., Castle J.D., Casanova J.E., Schwartz M.A. (2007). Arf6 and microtubules in adhesion-dependent trafficking of lipid rafts. Nat. Cell Biol..

[bib5] Bass M.D., Morgan M.R., Humphries M.J. (2007). Integrins and syndecan-4 make distinct, but critical, contributions to adhesion contact formation. Soft Matter.

[bib6] Bass M.D., Roach K.A., Morgan M.R., Mostafavi-Pour Z., Schoen T., Muramatsu T., Mayer U., Ballestrem C., Spatz J.P., Humphries M.J. (2007). Syndecan-4-dependent Rac1 regulation determines directional migration in response to the extracellular matrix. J. Cell Biol..

[bib7] Bass M.D., Morgan M.R., Roach K.A., Settleman J., Goryachev A.B., Humphries M.J. (2008). p190RhoGAP is the convergence point of adhesion signals from α5β1 integrin and syndecan-4. J. Cell Biol..

[bib8] Bass M.D., Morgan M.R., Humphries M.J. (2009). Syndecans shed their reputation as inert molecules. Sci. Signal..

[bib9] Bass M.D., Williamson R.C., Nunan R.D., Humphries J.D., Byron A., Morgan M.R., Martin P., Humphries M.J. (2011). A syndecan-4 hair trigger initiates wound healing through caveolin- and RhoG-regulated integrin endocytosis. Dev. Cell.

[bib10] Brunton V.G., MacPherson I.R., Frame M.C. (2004). Cell adhesion receptors, tyrosine kinases and actin modulators: a complex three-way circuitry. Biochim. Biophys. Acta.

[bib11] Caswell P.T., Chan M., Lindsay A.J., McCaffrey M.W., Boettiger D., Norman J.C. (2008). Rab-coupling protein coordinates recycling of α5β1 integrin and EGFR1 to promote cell migration in 3D microenvironments. J. Cell Biol..

[bib12] Caswell P.T., Vadrevu S., Norman J.C. (2009). Integrins: masters and slaves of endocytic transport. Nat. Rev. Mol. Cell Biol..

[bib13] Danen E.H., Sonneveld P., Brakebusch C., Fassler R., Sonnenberg A. (2002). The fibronectin-binding integrins α5β1 and αvβ3 differentially modulate RhoA-GTP loading, organization of cell matrix adhesions, and fibronectin fibrillogenesis. J. Cell Biol..

[bib14] Danen E.H., van Rheenen J., Franken W., Huveneers S., Sonneveld P., Jalink K., Sonnenberg A. (2005). Integrins control motile strategy through a Rho-cofilin pathway. J. Cell Biol..

[bib15] Dovas A., Yoneda A., Couchman J.R. (2006). PKCβ-dependent activation of RhoA by syndecan-4 during focal adhesion formation. J. Cell Sci..

[bib16] D’Souza-Schorey C., Chavrier P. (2006). ARF proteins: roles in membrane traffic and beyond. Nat. Rev. Mol. Cell Biol..

[bib17] D’Souza-Schorey C., Li G., Colombo M.I., Stahl P.D. (1995). A regulatory role for ARF6 in receptor-mediated endocytosis. Science.

[bib18] Dunphy J.L., Moravec R., Ly K., Lasell T.K., Melancon P., Casanova J.E. (2006). The Arf6 GEF GEP100/BRAG2 regulates cell adhesion by controlling endocytosis of β1 integrins. Curr. Biol..

[bib19] Geiger B., Bershadsky A., Pankov R., Yamada K.M. (2001). Transmembrane crosstalk between the extracellular matrix and the cytoskeleton. Nat. Rev. Mol. Cell Biol..

[bib20] Hu K., Ji L., Applegate K.T., Danuser G., Waterman-Storer C.M. (2007). Differential transmission of actin motion within focal adhesions. Science.

[bib21] Koo B.K., Jung Y.S., Shin J., Han I., Mortier E., Zimmermann P., Whiteford J.R., Couchman J.R., Oh E.S., Lee W. (2006). Structural basis of syndecan-4 phosphorylation as a molecular switch to regulate signaling. J. Mol. Biol..

[bib22] Miller M.L., Jensen L.J., Diella F., Jørgensen C., Tinti M., Li L., Hsiung M., Parker S.A., Bordeaux J., Sicheritz-Ponten T. (2008). Linear motif atlas for phosphorylation-dependent signaling. Sci. Signal..

[bib23] Morgan M.R., Humphries M.J., Bass M.D. (2007). Synergistic control of cell adhesion by integrins and syndecans. Nat. Rev. Mol. Cell Biol..

[bib24] Morgan M.R., Byron A., Humphries M.J., Bass M.D. (2009). Giving off mixed signals—distinct functions of α5β1 and αvβ3 integrins in regulating cell behaviour. IUBMB Life.

[bib25] Murakami M., Elfenbein A., Simons M. (2008). Non-canonical fibroblast growth factor signalling in angiogenesis. Cardiovasc. Res..

[bib26] Muralidharan-Chari V., Hoover H., Clancy J., Schweitzer J., Suckow M.A., Schroeder V., Castellino F.J., Schorey J.S., D’Souza-Schorey C. (2009). ADP-ribosylation factor 6 regulates tumorigenic and invasive properties in vivo. Cancer Res..

[bib27] Ott V.L., Rapraeger A.C. (1998). Tyrosine phosphorylation of syndecan-1 and -4 cytoplasmic domains in adherent B82 fibroblasts. J. Biol. Chem..

[bib28] Pellinen T., Arjonen A., Vuoriluoto K., Kallio K., Fransen J.A., Ivaska J. (2006). Small GTPase Rab21 regulates cell adhesion and controls endosomal traffic of β1-integrins. J. Cell Biol..

[bib29] Playford M.P., Schaller M.D. (2004). The interplay between Src and integrins in normal and tumor biology. Oncogene.

[bib30] Powelka A.M., Sun J., Li J., Gao M., Shaw L.M., Sonnenberg A., Hsu V.W. (2004). Stimulation-dependent recycling of integrin β1 regulated by ARF6 and Rab11. Traffic.

[bib31] Puklin-Faucher E., Sheetz M.P. (2009). The mechanical integrin cycle. J. Cell Sci..

[bib32] Ridley A.J., Schwartz M.A., Burridge K., Firtel R.A., Ginsberg M.H., Borisy G., Parsons J.T., Horwitz A.R. (2003). Cell migration: integrating signals from front to back. Science.

[bib33] Roberts M., Barry S., Woods A., van der Sluijs P., Norman J. (2001). PDGF-regulated rab4-dependent recycling of αvβ3 integrin from early endosomes is necessary for cell adhesion and spreading. Curr. Biol..

[bib34] Roca-Cusachs P., Gauthier N.C., Del Rio A., Sheetz M.P. (2009). Clustering of α5β1 integrins determines adhesion strength whereas αvβ3 and talin enable mechanotransduction. Proc. Natl. Acad. Sci. USA.

[bib35] Sandilands E., Frame M.C. (2008). Endosomal trafficking of Src tyrosine kinase. Trends Cell Biol..

[bib36] Santy L.C. (2002). Characterization of a fast cycling ADP-ribosylation factor 6 mutant. J. Biol. Chem..

[bib37] Santy L.C., Casanova J.E. (2001). Activation of ARF6 by ARNO stimulates epithelial cell migration through downstream activation of both Rac1 and phospholipase D. J. Cell Biol..

[bib38] Streuli C.H., Akhtar N. (2009). Signal co-operation between integrins and other receptor systems. Biochem. J..

[bib39] Sulka B., Lortat-Jacob H., Terreux R., Letourneur F., Rousselle P. (2009). Tyrosine dephosphorylation of the syndecan-1 PDZ binding domain regulates syntenin-1 recruitment. J. Biol. Chem..

[bib40] White D.P., Caswell P.T., Norman J.C. (2007). αvβ3 and α5β1 integrin recycling pathways dictate downstream Rho kinase signaling to regulate persistent cell migration. J. Cell Biol..

[bib41] Woods A., Couchman J.R., Johansson S., Höök M. (1986). Adhesion and cytoskeletal organisation of fibroblasts in response to fibronectin fragments. EMBO J..

[bib42] Worth D.C., Hodivala-Dilke K., Robinson S.D., King S.J., Morton P.E., Gertler F.B., Humphries M.J., Parsons M. (2010). αvβ3 integrin spatially regulates VASP and RIAM to control adhesion dynamics and migration. J. Cell Biol..

[bib43] Zimmermann P., Zhang Z., Degeest G., Mortier E., Leenaerts I., Coomans C., Schulz J., N’Kuli F., Courtoy P.J., David G. (2005). Syndecan recycling [corrected] is controlled by syntenin-PIP2 interaction and Arf6. Dev. Cell.

