# Supplementary material for: Syndecan-4 Phosphorylation Is a Control Point for Integrin Recycling
Source: Dev Cell. 2013 Mar 11;24(5):472–85. doi: 10.1016/j.devcel.2013.01.027 (PMC3605578; doi:10.1016/j.devcel.2013.01.027)
Supplement: Document S1. Figures S1–S6 and Supplemental Experimental Procedures [file mmc1.pdf]

Developmental Cell, Volume *24*

Supplemental Information

Syndecan-4 Phosphorylation

Is a Control Point for Integrin Recycling

Mark R. Morgan, Hellyeh Hamidi, Mark D. Bass, Stacey Warwood, Christoph Ballestrem, and Martin J. Humphries

Supplemental Information Inventory

Supplemental Figure S1: c-Src directly phosphorylates syndecan-4, related to Fig 1

Supplemental Figure S2: Phosphorylation-competence of syndecan-4 Y180 regulates FA turnover and cell migration, related to Fig 3

Supplemental Figure S3: Syndecan-4 phosphorylation regulates heterodimer-specific integrin localisation, related to Fig 5

Supplemental Figure S4: Syndecan-4 and Src regulate Arf6 activity, related to Fig 6

Supplemental Figure S5: Arf6 differentially regulates integrin recycling and membrane targeting, related to Fig 6

Supplemental Figure S6: Syndecan-4-mediated Arf6 activity regulates FA dynamics and cell migration, related to Fig 7

Supplemental Experimental Procedures

Fig S1 Morgan et al 2013

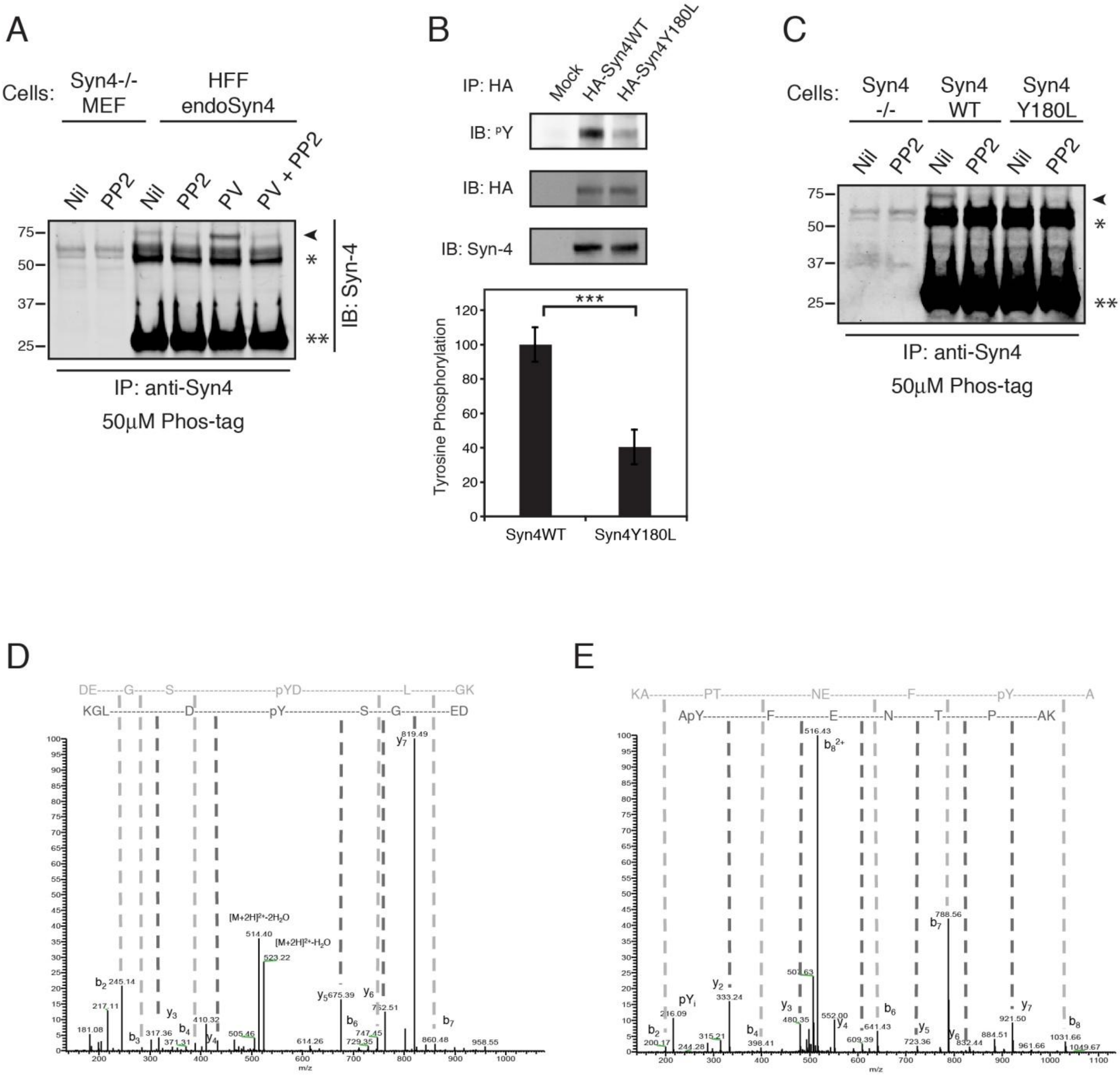

Supplemental Figure S1: c-Src directly phosphorylates syndecan-4, related to Fig 1

(A) Phosphorylation of endogenous syndecan-4 assessed by Phos-tag immunoblotting, following immunoprecipitation from human fibroblasts treated with or without pervanadate (PV) or PP2. Syndecan-4 null MEFs were used as a negative control. Blots from Fig 1B showing both syndecan-4 monomeric and dimeric bands; single asterisk denotes dimeric syndecan-4, double asterisk denotes monomeric syndecan-4 band and arrowhead denotes slow-migrating phosphorylated syndecan-4 band. Mean proportion of dimeric syndecan-4 that is phosphorylated (n=3): Nil 2.63%, PP2 0.72%, PV 11.70%, PV + PP2 1.84%. NB: Apparent molecular weights relate only to the size of unphosphorylated proteins and are approximate as Phos-tag distorts the migration of molecular weight markers. **(B)** Tyrosine phosphorylation of HA-Syn4WT and HA-Syn4Y180L expressed in HEK-293T cells and isolated by immunoprecipitation and assessed by anti-phospho-tyrosine immunodetection. Tyrosine phosphorylation was expressed relative to total HA detection. Data show normalised means ± SEM (*** $P<0.001$; Student's *t*-test.). **(C)** Phos-tag immunoblot of syndecan-4 isolated from pervanadate-treated Syn4WT and Syn4Y180L cells in the presence or absence of PP2. Blots from Fig 1D showing both monomeric and dimeric bands. Mean proportion of dimeric syndecan-4 that is phosphorylated (n=3): Syn4WT PV 13.83%, Syn4WT PV + PP2 5.13%, Syn4Y180L PV 6.55%, Syn4Y180L PV + PP2 3.23%. NB: Apparent molecular weights relate only to the size of unphosphorylated proteins and are approximate as Phos-tag distorts the migration of molecular weight markers. **(D, E)** Product ion spectra for phosphopeptides DEGSpYDLGK **(D)** and KAPTNEFpYA **(E)** derived from recombinant syndecan-4 cytoplasmic domain following phosphorylation by recombinant active c-Src. Annotations include b and y ions supporting the location of Y180 and Y197 phosphorylation.

Fig S2 Morgan et al 2013

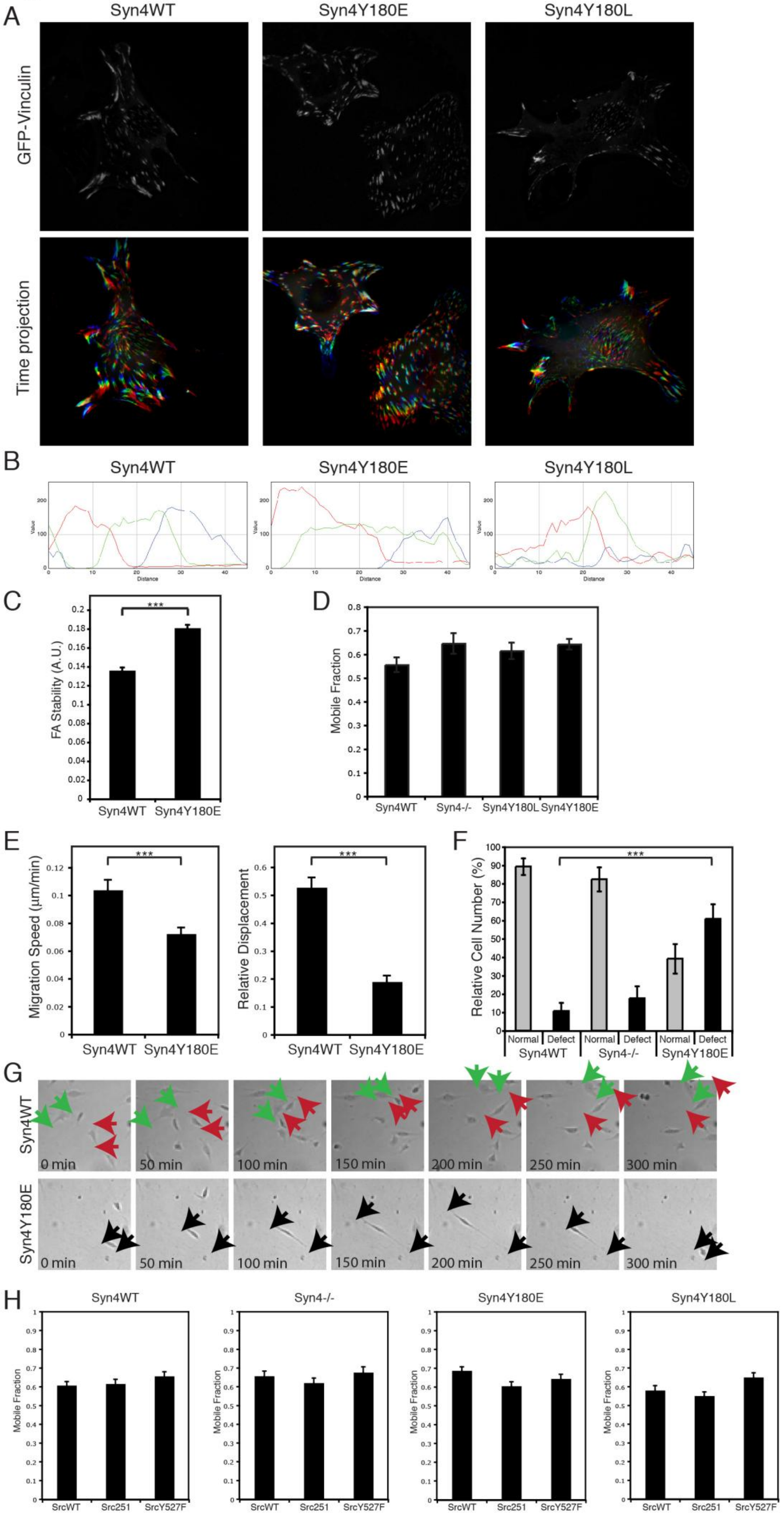

Supplemental Figure S2: Phosphorylation-competence of syndecan-4 Y180 regulates FA turnover and cell migration, related to Fig 3

Syn4WT-, Syn4Y180L-, Syn4Y180E-expressing cells were transfected with GFP-vinculin and FA translocation monitored by fluorescent live-cell imaging. **(A)** Red green blue (RGB) time projections of FA translocation of representative cells. Upper panel shows GFP-vinculin localisation in the 1st frame (shown in red in Fig. 3A). Lower panel shows colour-coded time projection images demonstrating FA movement over 18 frames (127.5 mins) – Frames 1-6: red, frames 7-12: green and frames 13-18: Blue. **(B)** RGB profiles calculated for representative FAs in RGB time projections of GFP-vinculin translocation in Syn4WT, Syn4Y180L, Syn4Y180E cells. The colour represents time (Red = 0-37.5 mins, Green = 45-82.5 mins, Blue = 90-127.5 mins), x-axis represents position (9.26μm length) and y-axis represents RGB signal intensity. Thus levels of positional colocalisation of red, green or blue inversely correlate with the speed of FA translocation. Consistent with Supplemental Movie S2 and the kymograph and tracking analyses in Figure 2B and C, Syn4Y180E FAs, compared to Syn4WT cells, exhibit high levels of RGB colocalisation demonstrating relatively static FAs, whereas Syn4Y180L cells have low levels of RGB colocalisation indicating fasts rates of translocation and reduced FA lifetimes. **(C)** Interference reflection microscopy was used to assess dynamics of endogenous FAs in Syn4WT and Syn4Y180E. Graph shows mean levels of positional colocalisation of FAs between 15 min time frames over an 8 hour time-course (error bars represent SEM) $P = 5.67 \times 10^{-5}$; Student's *t*-test ($n > 13$). Data are representative of 3 independent experiments. **(D)** Syn4WT, Syn4-/-, Syn4Y180L and Syn4Y180E cells were transfected with GFP-vinculin and FA component dynamics assessed by GFP-vinculin FRAP. Mean GFP-vinculin mobile fraction is shown (error bars represent SEM). Compare with GFP-vinculin half-time of recovery in Figure 4D. **(E)** Migration of Syn4WT and Syn4Y180E cells plated on CDMs was analysed over 17 hrs and 40 min by time-lapse microscopy. Migration speed (μm/min) and relative displacement (linear displacement/total distance migrated) are shown ± SEM (n = 43-70) *** P<0.001 (*Z*-test). **(F)** Migration of Syn4WT and Syn4Y180E on CDMs was analysed over 34 hrs and the frequency of tail retraction defects scored manually (means ± SEM are shown). $n > 400$; *** P<0.001 (Student's *t*-test). 50.1% ± 6.2% of the Syn4Y180E cells that exhibited a tail retraction defect migrated along the fibrillar ECM and recoiled back to their starting position (example shown in **G**). **(H)** Syn4WT, Syn4-/-, Syn4Y180L and Syn4Y180E cells were co-transfected with GFP-vinculin and either SrcWT, SrcY527 or Src251 and FA component dynamics assessed by GFP-vinculin FRAP. Mean GFP-vinculin mobile fraction is shown (error bars represent SEM). Compare with GFP-vinculin half-time of recovery in Figure 4D.

Fig S3 Morgan et al 2013

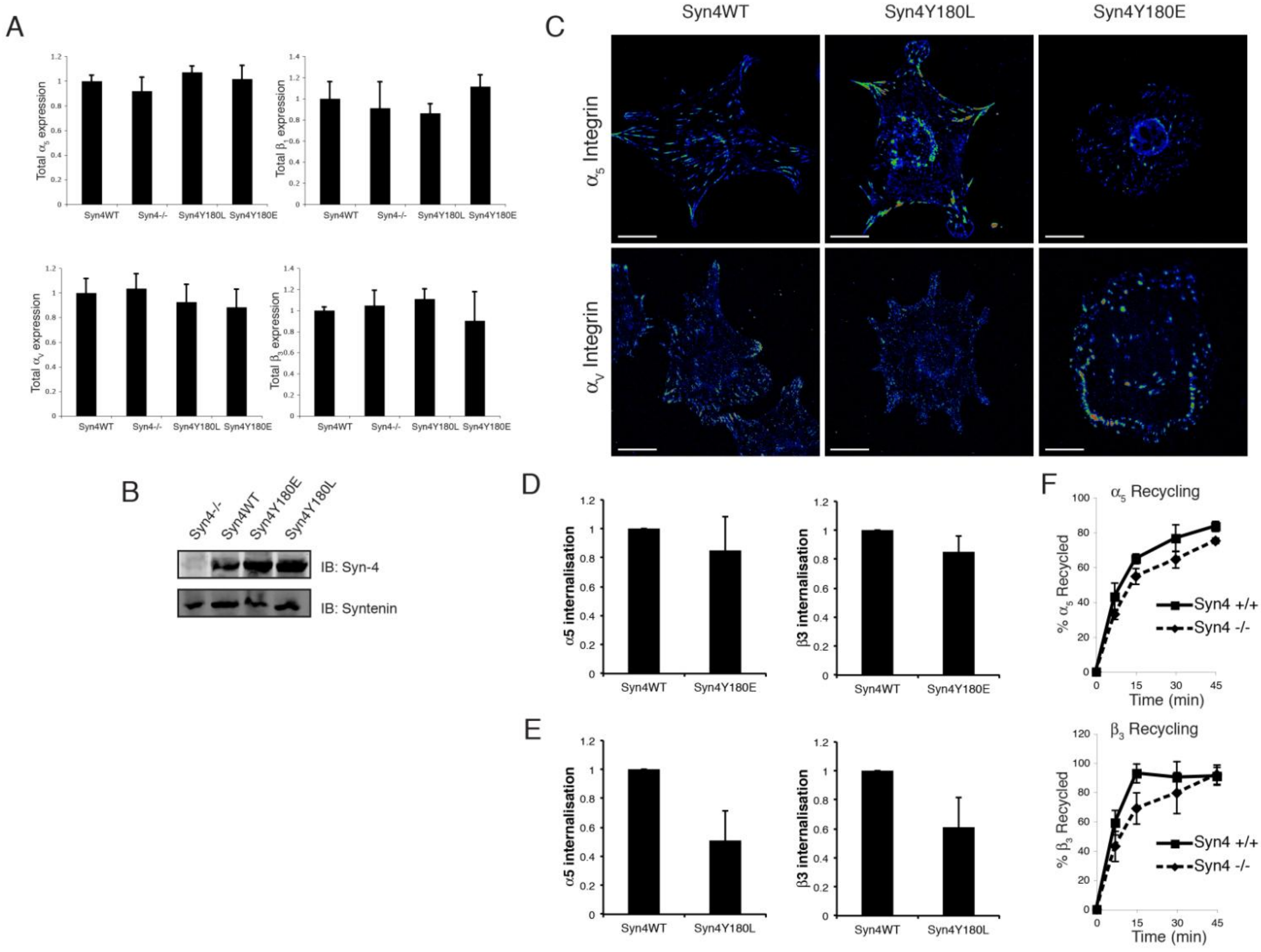

Supplemental Figure S3: Syndecan-4 phosphorylation regulates heterodimer-specific integrin localisation, related to Fig 5

(A) Densitometric quantitation of α5, β1, αV and β3 integrin expression detected by immunoblotting Syn4WT, Syn4-/-, Syn4Y180L and Syn4Y180E total cell lysates (Fig. 5A). Integrin subunit expression levels were calculated relative to actin and normalised to levels in Syn4WT cells. Graphs show means from 2 or 3 independent experiments ± standard deviation. **(B)** Expression of syndecan-4 in Syn4-/-, Syn4WT, Syn4Y180E and Syn4Y180L total cell lysates. Syntenin was used as a loading control. **(C)** The dynamic range of α5 and αV integrin immunofluorescence intensity in Syn4WT, Syn4Y180L and Syn4Y180E MEFs (standard representative images presented in Fig. 5D). Rainbow RGB look up tables were applied to immunofluorescence micrographs using ImageJ. All images were acquired using the same settings and were treated in exactly the same way. Scale bars = 20μm. **(D,E)** Levels of α5 and β3 internalisation in cells expressing SynWT and SynY180E **(D)** or Syn4Y180L **(E)**. Data are means ± SEM of 3 independent experiments. **(F)** Recycling of α5, β3 and αV integrin subunits was assessed in wild type cells expressing endogenous syndecan-4 (Syn4 +/+) and or syndecan-4 deficient cells (Syn4 -/-). Data are means ± standard deviation.

Fig S4 Morgan et al 2013

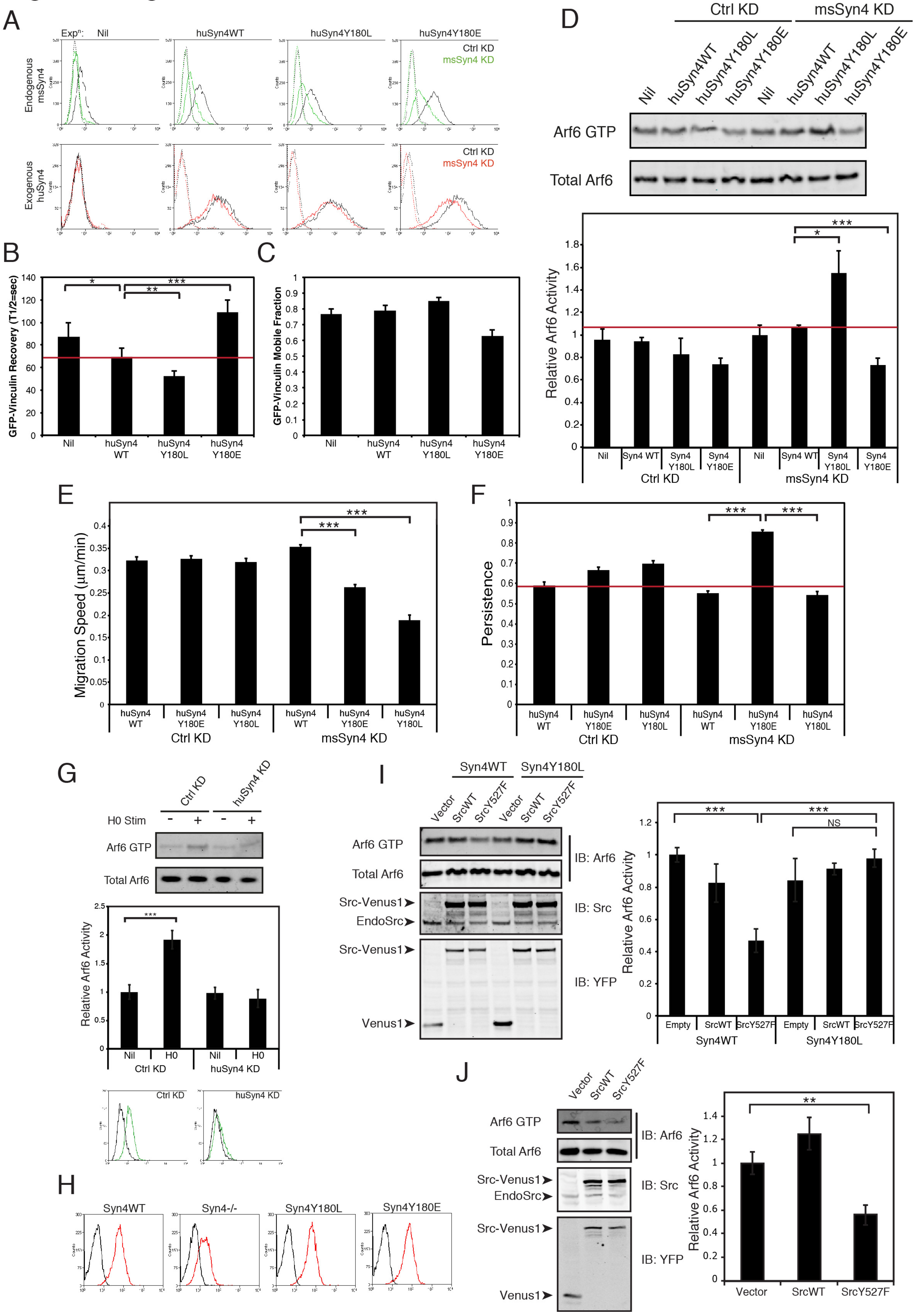

Supplemental Figure S4: Syndecan-4 and Src regulate Arf6 activity, related to Fig 6

(A) Flow cytometry of NIH-3T3-derived cells, which endogenously express wild-type mouse syndecan-4 (msSyn4), stably expressing human syndecan-4 constructs (huSyn4WT, huSyn4Y180L, or huSyn4Y180E) or no construct (Nil), transiently transfected with non-targeting siRNA (Ctrl KD; black lines) or mouse syndecan-4-targeting siRNA (msSyn4 KD; green or red lines). Endogenous mouse syndecan-4 detected with KY8,2 and Alexa Fluor 488 (top panel); exogenous human syndecan-4 constructs detected with 5G9 and Alexa Fluor 647 (lower panel). Dotted lines show isotype-matched IgG controls. Species-specific antibodies were used to detect endogenous and exogenous syndecan-4 simultaneously in the same cells (using secondary antibodies with different emission spectra and intensities). **(B, C)** FRAP analysis of GFP-vinculin in NIH-3T3-derived cells, expressing huSyn4WT, huSyn4Y180L, huSyn4Y180E or no construct (Nil), following siRNA-mediated suppression of endogenous mouse syndecan-4. Mean GFP-vinculin recovery half-time **(B)** and mobile fraction **(C)** are shown. Red line represents vinculin recovery in huSyn4WT-expressing cells *n*=93-100 FAs per condition; *** $P<0.001$, ** $P<0.01$, * $P<0.05$ (Student's *t*-test). Data are means ± SEM of 2 independent experiments. **(D)** Steady-state Arf6 activity (Arf6 GTP) assessed by effector pull-down in NIH-3T3-derived cells, expressing huSyn4WT, huSyn4Y180L, huSyn4Y180E or no construct (Nil), following either Ctrl KD or endogenous msSyn4 KD. Red line represents Arf6 activity in huSyn4WT following msSyn4 KD. **(E, F)** Migration of in NIH-3T3-derived cells, expressing huSyn4WT, huSyn4Y180L, huSyn4Y180E, transiently transfected with non-targeting siRNA (Ctrl KD) or mouse syndecan-4-targeting siRNA (msSyn4 KD), in scratch wound assays. Individual cells were tracked over 16 hrs. Migration speed **(E)** and directional persistence **(F)** are shown. Red line represents directional persistence in huSyn4WT-expressing cells following Ctrl KD. Values are means ± SEM $n>50$ cells per condition (*** $P<0.001$; *Z*-test). **(G)** Arf6 activity in response to syndecan-4 engagement was assessed by plating human fibroblasts, transfected with either control siRNA (Ctrl) or human Syndecan-4-targeting siRNA (huSyn4 KD), on the central cell-binding domain of fibronectin, Fn6-10, and stimulating with a soluble heparin-binding domain containing fragment of fibronectin, H0. Arf6 activity was analysed by effector pull-down and quantitative immunoblotting. Flow cytometric analysis shows syndecan-4 expression (Black lines = isotype-matched IgG control, Green lines = Endogenous human syndecan-4). **(H)** Flow cytometric analysis of Alexa Fluor 647-conjugated H0-binding to Syn4WT, Syn4-/-, Syn4Y180L and Syn4Y180E cells. Black line = unlabelled cells, Red line = H0-647. **(I)** Steady-state Arf6 activity (Arf6 GTP) assessed by effector pull-down in Syn4WT and Syn4Y180L cells expressing Venus1 (Vector), SrcWT-Venus1 or SrcY527F-Venus1. **(J)** Steady-state Arf6 activity (Arf6 GTP) assessed by effector pull-down in A2780 ovarian carcinoma cells expressing Venus1 (Vector), SrcWT-Venus1 or SrcY527F-Venus1. **(I, J)** Expression of the constructs was determined by immunoblotting (IB) for Src and YFP. Src-Venus1 arrow highlights Src-Venus1 construct bands, EndoSrc arrow identifies the endogenous Src bands and Venus-1 arrow identifies the Venus1 construct encoded by the empty vector. Graphs show mean Arf6 activity, normalised to total Arf6, for and 3 **(D, J)** or 6 **(G, I)** independent experiments ± SEM (*** $P<0.001$; ** $P<0.01$, * $P<0.05$, NS = not significant; *P* values calculated with Student's *t*-test).

Fig S5 Morgan et al 2013

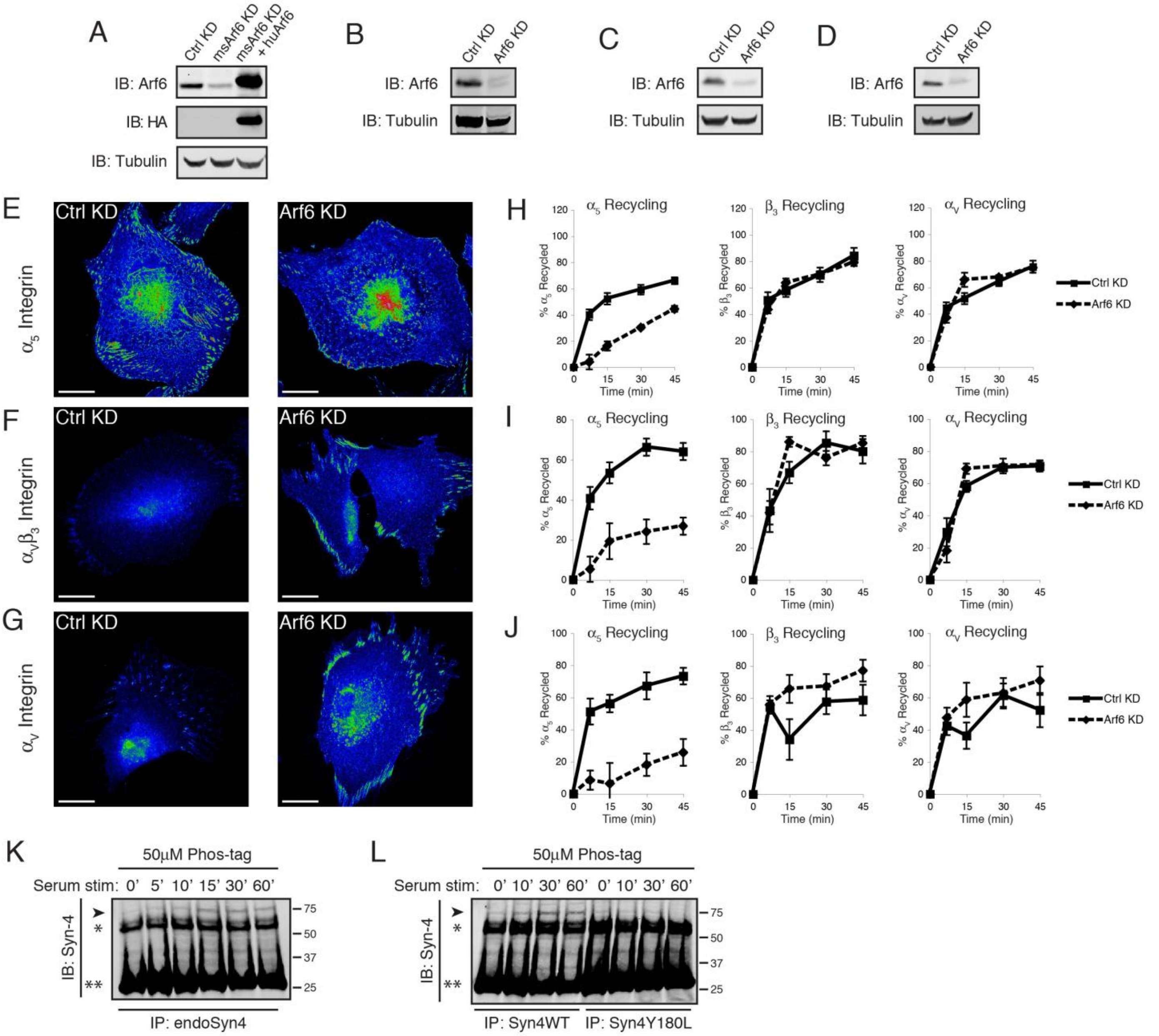

Supplemental Figure S5: Arf6 differentially regulates integrin recycling and membrane targeting, related to Fig 6 **(A)** Arf6 expression determined by immunoblotting for Arf6 and HA in NIH-3T3 cells following transfection with control oligonucleotide (Ctrl KD), mouse Arf6-targeting siRNA oligonucleotide (msArf6 KD) or mouse Arf6-targeting oligonucleotide and HA-tagged human Arf6 cDNA (msArf6 KD + huArf6). **(B-D)** Arf6 expression was assessed in Ctrl KD and Arf6 KD A2780 cells **(B)**, A375-SM cells **(C)** and MEFs **(D)**. **(E-G)** The dynamic range of α5 **(E)**, αVβ3 **(F)** and αV **(G)** integrin immunofluorescence intensity in human fibroblasts plated on fibronectin following siRNA-mediated suppression of Arf6 activity (Arf6 KD) relative to cells transfected with control oligonucleotide (Ctrl KD) (standard representative images presented in Fig. **6D-F**). Rainbow RGB look up tables were applied to immunofluorescence micrographs using ImageJ. All images were acquired using the same settings and were analysed in exactly the same way. Scale bars = 30μm. **(H)** Recycling of α5, β3 and αV integrin subunits was assessed in Arf6 KD and Ctrl KD human fibroblasts. **(I)** Integrin recycling in A2780 ovarian carcinoma cells following transfection with control oligonucleotides (Ctrl KD) human Arf6-targeting siRNA oligonucleotides (Arf6 KD). **(J)** Integrin recycling in A375-SM malignant melanoma cells following transfection with control oligonucleotides (Ctrl KD) human Arf6-targeting siRNA oligonucleotides (Arf6 KD). Data show means ± SEM of 3 independent experiments. **(K, L)** Phosphorylation of endogenous syndecan-4 **(K)** or Syn4WT and Syn4Y180L **(L)** assessed by Phos-tag immunoblotting, following immunoprecipitation from cells stimulated with 20% serum. Blots from Fig 1I/J showing both monomeric and dimeric bands; asterisk denotes dimeric syndecan-4, arrowhead denotes slow-migrating phosphorylated syndecan-4 band. Mean proportion of dimeric syndecan-4 that is phosphorylated (n=3): **(K)** endoSyn4 0 min = 5.02%, 30 min = 13.09% **(L)** Syn4WT 0 min = 5.43%, Syn4WT 30 min = 18.61%, Syn4Y180L 0 min = 6.00%, Syn4Y180L 30 min = 5.97%. NB: For Phos-tag immunoblots, the apparent molecular weights relate only to the size of unphosphorylated proteins and are approximate as Phos-tag distorts the migration of molecular weight markers.

Fig S6 Morgan et al 2013

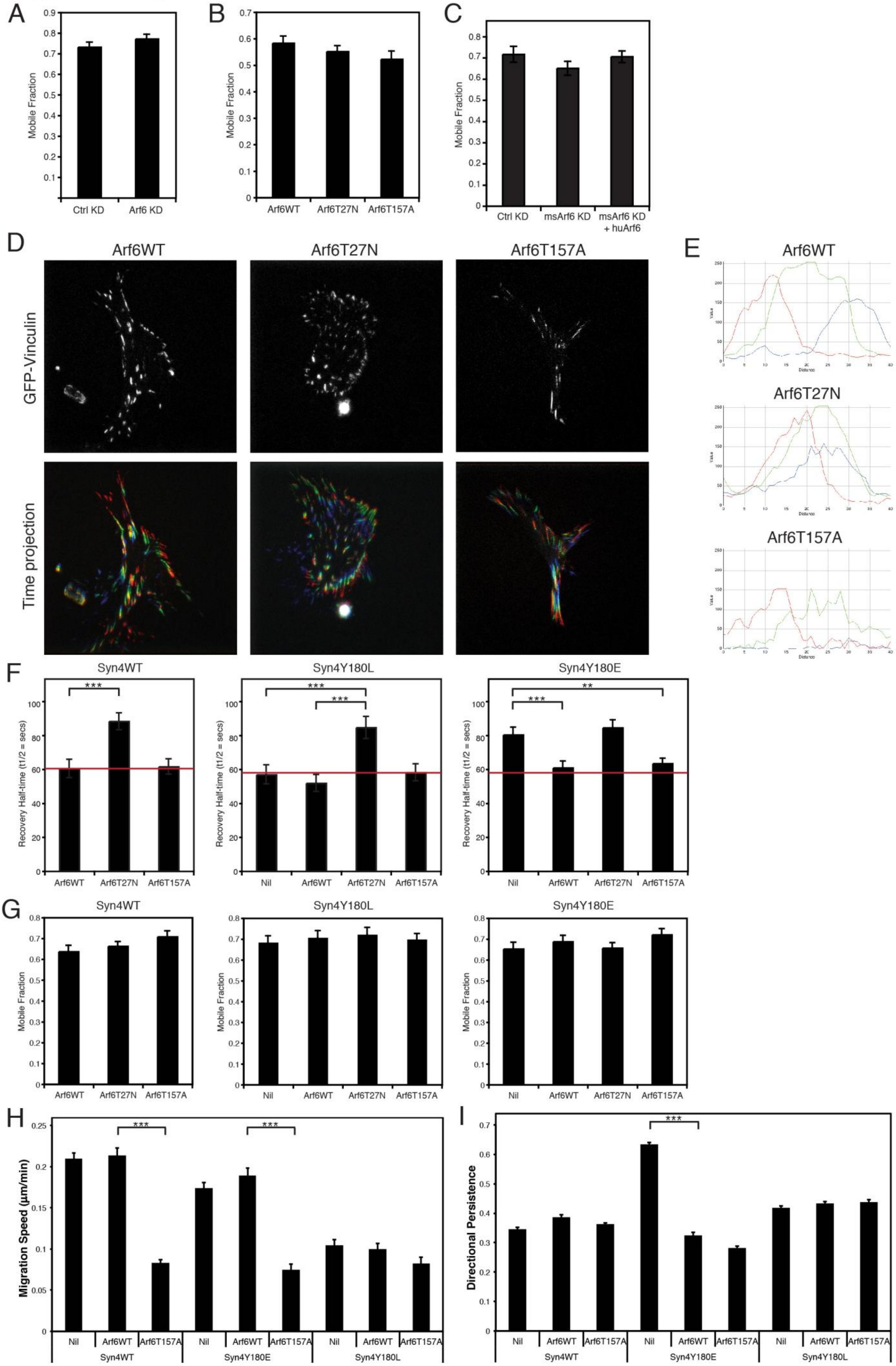

Supplemental Figure S6: Syndecan-4-mediated Arf6 activity regulates FA dynamics and cell migration, related to Fig 7

(A) Arf6 KD and Ctrl KD (Fig. 7A) cells were transfected with GFP-vinculin and FRAP assessed. **(B)** GFP-vinculin FRAP in wild-type MEFs, expressing endogenous syndecan-4, co-transfected with Arf6 constructs (wild-type Arf6WT, dominant negative Arf6T27N or fast-cycling mutant Arf6T157A). **(C)** GFP-vinculin FRAP assessed in NIH-3T3 cells following transfection with control oligonucleotide (Ctrl KD), mouse Arf6-targeting siRNA oligonucleotide (msArf6 KD) or mouse Arf6-targeting oligonucleotide and human Arf6 cDNA (msArf6 KD + huArf6). For all FRAP experiments, mean GFP-vinculin mobile fraction is shown (error bars represent SEM). Compare with GFP-vinculin half-time of recovery in Figure 7A-C. **(D,E)** NIH-3T3 cells were co-transfected with GFP-vinculin and either Arf6WT, Arf6T27N or Arf6T157A and FA translocation monitored by live-cell TIRF imaging. **(D)** Red green blue (RGB) time projections of FA translocation of representative cells. Upper panel shows GFP-vinculin localisation in the 1st frame (shown in red in Fig. 7D). Lower panel shows colour-coded time projection images demonstrating FA movement over 15 frames (105 mins) – Frames 1-5: red, frames 6-10: green and frames 11-15: Blue. **(E)** RGB profiles calculated for representative FAs in RGB time projections of GFP-vinculin translocation in Arf6WT, Arf6T27N or Arf6T157A-expressing cells. The colour represents time (Red = 0-30 mins, Green = 37.5-67.5 mins, Blue = 75-105 mins), x-axis represents position (8.044μm length) and y-axis represents RGB signal intensity. Levels of positional colocalisation of red, green or blue inversely correlate with the speed of FA translocation. Consistent with Supplemental Movie S3 and the tracking analyses in Figure 7E, FAs in Arf6T27N cells, compared with Arf6WT cells, exhibit high levels of RGB colocalisation demonstrating relatively static FAs. By contrast, Arf6T157A cells have low levels of RGB colocalisation signifying fast rates of translocation and short FA lifetimes. **(F,G)** Syn4WT, Syn4Y180L and Syn4Y180E cells were co-transfected with GFP-vinculin and either Arf6WT, Arf6T27N or Arf6T157A and FA component dynamics assessed by GFP-vinculin FRAP. Mean GFP-vinculin recovery half-time is shown ± SEM (*n*=36-81) *** *P*<0.001, ** *P*<0.01 (Student's *t*-test). Red line represents rate of vinculin recovery in Syn4WT cells expressing Arf6WT. **(F)**. GFP-vinculin mobile fraction is shown ± SEM **(G)**. **(H, I)** Migration of Syn4WT, Syn4Y180E and Syn4Y180L cells, co-transfected with GFP-vinculin and either Arf6WT, Arf6T157A or no other cDNA (Nil), on 2D fibronectin analysed over 16 hrs by time-lapse microscopy. Migration speed **(H)** and directional persistence **(I)** are shown. Values are means ± SEM (*n* = 42-98). *** *P*<0.001 (Student's *t*-test).

SUPPLEMENTAL EXPERIMENTAL PROCEDURES

Antibodies and reagents

The following primary antibodies were used in this study: anti-mouse α5 integrin monoclonal (5H10-27; BD Biosciences); anti-α5 integrin (H-104; Santa Cruz); anti-mouse αV integrin (C8F12; gift from Charles Streuli); anti-mouse αV integrin (RMV-7; Cymbus Biotechnology and BD Biosciences); anti-mouse β3 (2C9.G2; BD Biosciences); anti-β3 integrin (Cell Signaling Technology); anti-mouse β1 integrin (9EG7; gift from D. Vestweber); anti-human α5 integrin (mab11; gift from K. Yamada); anti-human α5 integrin (VC5; BD Biosciences); anti-human αV integrin (L230; ATCC); anti-human αVβ3 integrin (LM609; Millipore); anti-human β3 integrin (B3A; Millipore); anti-mouse syndecan-4 (KY8.2; BD Biosciences); anti-human syndecan-4 (5G9; Santa Cruz); anti-syndecan-4 (3644; BioVision); anti-Src (GD11; Millpore); anti-GFP (Living Colours A.v. polyclonal; BD Biosciences) anti-HA (HA-7; Sigma); anti-HA (MSA106; Stressgen); Unlabelled and HRP-conjugated anti-HA (Y-11; Santa Cruz); anti-phosphotyrosine (4G10; Millipore); HRP-conjugated anti-phosphotyrosine (4G10 Platinum-HRP; Millipore); biotinylated anti-phosphotyrosine Fab' fragment (RC20-Biotin; Transduction Laboratories); anti-Arf6 (ARFAG; Sigma-Aldrich); anti-syntenin/SDCBP (S1701; Sigma-Aldrich); anti-mitochondrial HSP70 (JG1; Affinity BioReagents); anti-actin (AC-40; Sigma-Aldrich); anti-tubulin (DM1A; Sigma-Aldrich). Anti–mouse, anti-rabbit and anti-rat IgG conjugated to Cy2 (Jackson ImmunoResearch) or IR800 (Rockland). Alexa Fluor 680–conjugated anti–mouse, anti-rat and anti-rabbit IgG, Alexa Fluor 680–conjugated Streptavidin and TRITC-conjugated phalloidin were obtained from Invitrogen. HRP-conjugated anti mouse was purchased from Dako Cytomation. Human plasma fibronectin was purchased from Sigma-Aldrich. Protein G Sepharose beads were purchased from Zymed and Glutathione Sepharose beads were from GEC Healthcare. PP2 and PP3 were obtained from Calbiochem. Phos-tag was from Wako Chemicals Gmbh. GFP-vinculin cDNA was a gift from B. Geiger. SrcWT, Src251 and SrcY527F constructs were gifts from H. Varmus and SrcWT-Venus1 (wild-type c-Src with a c-terminal Venus1 element) was a gift from C. Wellbrock. Arf6WT and Arf6T27N were gifts from P. Chavrier and Arf6T157A was a gift from L. Santy. Syntenin-GFP was a gift from P. Zimmermann. GST-Syn4WT was generated by cloning the syndecan-4 cytoplasmic domain into pGEX-4T1 (Invitrogen) using standard molecular biology techniques.

Syn4Y180L, Syn4Y180E, Syn4Y197E, GST-Syn4Y180L, GST-Syn4Y188L and SrcY527F-Venus1 constructs were generated using a QuickChange XLII site-directed mutagenesis kit (Stratagene), using either Syn4WT (Bass, 2007b), IL2R-Syn4WT, GST-Syn4WT or SrcWT-Venus1 as templates.

Cell culture

Immortalised wild-type and syndecan-4-/- mouse embryonic fibroblasts (MEFs) (Bass, 2007b) were grown at the large T-antigen permissive temperature of 33˚C in Dulbecco's modified Eagles medium (DMEM) (Sigma-Aldrich) supplemented with 10% fetal bovine serum (FBS), 2mM L-glutamine, and 20U/ml IFNγ (Sigma-Aldrich). Syn4WT, Syn4Y180L,

Syn4Y180E and Syn4Y197E cells were generated by retroviral transduction of wild-type and mutant (Y180L, Y180E and Y197E) human syndecan-4 constructs into syndecan-4-/- MEFs, as described previously (Bass, 2007b). Puromycin-resistant (5µg/ml) cells were subjected to fluorescence activated cell sorting to establish similar levels of cell-surface syndecan-4 expression. Puromycin-resistant Syn4-/- control cells were generated by infecting syndecan-4-/- MEFs with virions produced by AM-12 cells transfected with empty pBabePuro. Levels of syndecan-4 expression in Syn4WT cells was determined to be comparable to that in human fibroblasts expressing endogenous syndecan-4 by flow cytometry following non-enzymatic cell-dissociation (Mean fluorescence 25.57 and 29.56, respectively). Large T-antigen immortalised human dermal fibroblasts (TIFs) were grown in DME supplemented with 15% FBS and 2mM L-glutamine at 37˚C. NIH-3T3 MEFs and A375-SM malignant melanoma cells were cultured at 37˚C in DMEM supplemented with 10% FBS and 2mM L-glutamine. A2780 ovarian carcinoma cell lines were cultured in RPMI-1640 with 10% FBS and 2mM glutamine. A375-SM and A2780 cells both use $\alpha5\beta1$, $\alpha V\beta3$ and syndecan-4 to engage fibronectin (Appierto, 2007; Caswell, 2008; Gehlsen, 1992; Mostafavi-Pour, 2003)

Transfections

For FRAP experiments and live-cell imaging $1x10^6$ MEFs or NIH-3T3 cells were transiently transfected with 3µg plasmid DNA using the Amaxa Nucleofector system according to manufacturer's instructions using MEF2 supplement and program A-23. For syndecan-4 ELISA and immunoprecipitation, 293T cells were transfected with HA-tagged syndecan-4 constructs using polyethylenimine (PEI). Briefly, 81µl PEI (1mg/ml in 150mM NaCl) was added to 669µl DMEM and incubated at room temperature for 2 mins. 6µg DNA was added to 750µl DMEM. The PEI and DNA mixes were combined, vortexed for 2 mins and incubated at room temperature for 20 mins. Cells were incubated with DNA/PEI complexes for 16 hrs at 37°C. For recycling assays, NIH-3T3 cells were transfected with Arf6WT or Arf6T157A using Lipofectamine PLUS according to manufacturer's instructions. NIH-3T3, Syn4WT and Syn4Y180L cells were transfected with Venus1, SrcWT-Venus1 or SrcY527F-Venus1 using Lipofectemine PLUS for Arf6 activity assays. NIH-3T3, Syn4WT, Syn4Y180L and Syn4Y180E cells were co-transfected with Arf6WT or Arf6T157A and GFP-vinculin using Lipofectemine PLUS for migration assays, as were MEF cell lines for transfection of GFP-syntenin for co-immunoprecipitation experiments. A2780 ovarian carcinoma cells were transfected with Venus1, SrcWT-Venus1 or SrcY527F-Venus1 the Amaxa Nucleofector system using Kit T, supplement 1 and program A-23. siRNA duplexes targeting human Arf6 sequence (sense) 5′-CGGCAUUACUACACUGGGA-3′, mouse Arf6 sequence (sense) 5′-CUGACAUUUGACACGAAUA-3′, mouse syndecan-4 sequence (sense) 5′-CGAAGGCAGUUACGACUUG-3′ (ALL with on TARGET modification for enhanced specificity), and a non-targeting control duplex were obtained from Thermo Fisher Scientific. Silencer Select siRNA targeting human syndecan-4 sequence (sense) 5′-GCUAUGACCUGGGCAAGAATT-3′ and 5′-CUACUGCUCAUGUACCGUATT-3′ were obtained from Life Technologies and used together. Either Thermo Fisher siRNA duplexes targeting mouse syntenin sequence (sense) 5′-ACGCCUAAGAGUUGCCGUA-3′ or Mission esiRNA targeting SDCBP (EMU073201) were used to knockdown mouse syntenin expression. siRNA mediated-knockdown of Arf6 expression was achieved using

Lipofectamine 2000 according to manufacturer's instructions as described previously (Bass, 2008). siRNA-mediated knockdown of mouse or human syndecan-4 or mouse syntenin expression was achieved by using an Amaxa Nucleofector system according to manufacturer's instructions using NHDF or MEF2 kits and program A-23 and 300nM oligonucleotides. Levels of protein knockdown were assessed by western blotting or flow cytometry.

Solid-phase analysis of syndecan-4 tyrosine phosphorylation

1. Endogenous syndecan-4 phosphotyrosine ELISA

To assess tyrosine phosphorylation of endogenous syndecan-4, wild-type MEFs and syndecan-4-null MEFs were serum-starved for 1 hr at 37°C. To allow determination of levels of total syndecan-4, cells were surface labelled with 133μg/ml sulfo-NHS-SS-Biotin at 37°C for 15 mins, washed twice with warm DMEM and returned to 37°C for 15 mins to recover prior to further treatments. Cells were pre-incubated with pervanadate (3mM H_2O_2, 0.1mM vanadate) for 15 mins at 37°C then were treated with vehicle, PP2 (10μM) or PP3 (10μM) for 60 mins at 37°C in the presence of pervanadate. Cells were lysed and endogenous mouse syndecan-4 captured using plates coated with anti-mouse syndecan-4 (KY8.2). Tyrosine phosphorylation was detected using 4G10 Platinum-HRP and biotinylated syndecan-4 was detected using Extravidin-Peroxidase (Sigma-Aldrich). Plates were washed extensively and developed with ABTS (Bass, 2007a).
For each treatment, total syndecan-4 levels were calculated as total biotin signal minus the background biotin signal detected for syn-4-null MEFs. Levels of tyrosine phosphorylation were expressed relative to total syndecan-4 detection.

2. HA-syndecan-4 phosphotyrosine ELISA

HEK-293T cells expressing nil or HA-tagged syndecan-4 (HA-Syn4WT or HA-Syn4Y180L) were serum-starved for 1 hr at 37°C and incubated with pervanadate (3mM H_2O_2, 0.1mM vanadate) for 60 mins at 37°C prior to lysis. HA-Syn4 was immobilised with anti-HA (MSA-106) and tyrosine phosphorylation was detected using anti-phosphotyrosine (4G10) and anti-mouse-HRP. Total syndecan-4 was captured with anti-human syndecan-4 (5G9) and detected with anti-HA-HRP (Y11-HRP). Plates were washed extensively and developed with ABTS (Bass, 2007a). Specific HA-syndecan-4 phosphotyrosine and total HA-syndecan-4 signals were calculated by subtracting the background signal detected for untransfected HEK-293T cells. Levels of HA-Syn4 tyrosine phosphorylation were expressed relative to total syndecan-4 detection in the same sample.

HA-syndecan-4 immunoprecipitation

Untransfected, or HA-tagged syndecan-4 (HA-Syn4WT or HA-Syn4Y180L)-expressing, HEK-293T cells were pervanadate treated and lysed as described above. HA-Syn4 was immunoprecipitated from clarified pre-cleared lysate with 1μg anti-HA mAb (HA-7) and 45μl Protein G Sepharose beads (Zymed) for 1 hr at 4°C. Beads were washed 3 times with lysis buffer, washed twice in heparinase buffer (50mM Hepes pH6.5, 50mM NaOAc, 150mM NaCl, 5mM $CaCl_2$) and resuspended in heparinase buffer containing 250mU Heparinase I, 66.7mU Heparinase II and 8.3mU Heparinase III.

Samples were incubated at 37°C for 4 hrs with fresh enzymes added after 2 hrs. Proteins were eluted with reducing sample buffer, resolved by SDS-PAGE and detected by western blotting (anti-phosphotyrosine (4G10); anti-HA (MSA-106); anti-syndecan-4 (3644)).

Phos-tag gel electrophoresis of syndecan-4

HFFs, expressing endogenous syndecan-4, or Syn4-/-, Syn4WT and Syn4Y180L MEFs were treated in the presence or absence of pervanadate or PP2 as described above, or were serum-starved for 16 Hrs and stimulated with DMEM containing 20% FBS for 0, 5, 10, 15, 30 or 60 mins, prior to lysis (lysis buffer: 1% Triton-X100, 0.1% SDS, 0.5% sodium deoxycholate, 50mM Tris pH7.5, 100mM NaCl, 2mM $MgCl_2$, 10% glycerol). Syndecan-4 was immunoprecipitated from clarified lysate with 3-5µg anti-syndecan-4 mAb (5G9) and 60-80µl Protein G Sepharose beads for 1 hr at 4°C. Immune-complexes were resolved on SDS-PAGE gels containing 10% acrylamide, 50µM Phos-tag (Kinoshita, 2006) (Wako Chemicals Gmbh) and 100µM $MnCl_2$. Phos-tag was used according to manufacturers instructions. Following electrophoresis and prior to western blotting Phos-tag gels were subjected to 2 x10 min washes in transfer buffer containing 1mM EDTA followed by at least 2 x10 min washes in transfer buffer without EDTA. Syndecan-4 was immunodetected with Alexa Fluor 790-conjugated anti-syndecan-4 (3644).

Phos-tag binding to phosphorylated proteins allowed band-shift resolution of phosphorylated syndecan-4. The proportion of syndecan-4 that was phosphorylated was determined by densitometric quantitation of band intensity.

Metabolic labelling of recombinant syndecan-4 cytoplasmic domains

Purified GST-tagged or recombinant custom-synthesised (Cambridge Peptides) syndecan-4 cytoplasmic domain peptides were incubated with 43ng active recombinant Src (Stressgen), 50µM ATP and 5µCi [γ-^{33}P-ATP] (Perkin Elmer) in reaction buffer (25mM MOPs pH 7.2, 125mM β-glycerophosphate, 20mM $MgCl_2$, 12.5mM $MnCl_2$, 5 mM EGTA, 0.25 mM DTT) at 30˚C for the appropriate time. Reactions were stopped by addition of 3% phosphoric acid and proteins resolved by SDS-PAGE. Following fixation and coommassie staining, gels were dried and exposed to Fujifilm BAS Cassette 2040. Gels were scanned on Bio-Rad Phosphoimager and analysed using Quantity One 4.5.0 Molecular Imager FX software.

Mass spectrometry and data analysis

Recombinant custom-synthesised syndecan-4 cytoplasmic domain peptides were incubated with 43ng recombinant active Src (Stressgen) and 50µM ATP in reaction buffer (25mM MOPs pH 7.2, 125mM β-glycerophosphate, 20mM $MgCl_2$, 12.5mM $MnCl_2$, 5 mM EGTA, 0.25 mM DTT) at 30˚C. Syndecan-4 peptides were separated from the recombinant Src on Nanosep 10K Omega centrifugation columns (PALL Life Sciences). Samples were subjected to in-solution tryptic digestion. Digested samples were analysed by LC-MS/MS using a NanoAcquity LC (Waters) coupled to an LTQ Velos (Thermo Fisher Scientific) mass spectrometer. Peptides were selected for fragmentation automatically by data-dependent analysis. Data produced were searched using Mascot (Matrix Science UK), against the Swiss-Prot database.

Phosphorylation was included as a variable modification. The phosphorylation sites suggested were validated by manual inspection of product ion spectra.

Syndecan-4 Co-Immunoprecipitation

GST-syntenin-transfected cells were lysed in 0.5% Igepal buffer (0.5% Igepal CA-630, 10 mM Tris-HCl pH 7.5, 250 mM NaCl, 0.5 mM EGTA, 10% glycerol, 10 mM sodium fluoride, 5 mM sodium orthovanadate, 10 µg/ml leupeptin, 10 µg/ml aprotinin and 0.5 mM AEBSF) 24-72 Hrs post-transfection. Clarified lysate was incubated with anti-syndecan-4 (5G9) antibody-bound protein G sepharose beads for 1 Hr at 4°C. Beads were washed 3 times with lysis buffer and immune-complexes resolved by SDS/PAGE.

GST-Pull down

Confluent MEFs were lysed in RIPA buffer (50 mM Tris-HCl pH 7.4, 150 mM NaCl, 10 mM MgCl2, 5 mM EGTA, 1% (v/v) Triton X-100, 10 mM sodium fluoride, 5 mM sodium orthovanadate, 10 µg/ml leupeptin, 10 µg/ml aprotinin and 0.5 mM AEBSF). Clarified lysate was pre-cleared with GST-coated beads at 4°C for 30 min and incubated with GST-Syn4 (Syn4WT, Y180E, Y180L or Y197E) proteins-bound to glutathione sepharose beads for 2 Hr at 4°C. Beads were washed 3 times with lysis buffer and bound proteins resolved by SDS/PAGE.

Fluorescent live-cell imaging

Cells expressing GFP-vinculin were plated on glass-bottom 24-well plates, coated using 10µg/ml plasma fibronectin, 24-48 hours post-transfection, and imaged at 37°C in Ham's F12 medium supplemented with 2% FBS. Time-lapse epifluorescence images were acquired on an Olympus inverted microscope (IX71) using DeltaVisionRT software (Applied Precision) and a 60x/NA1.42 Plan ApoN objective. Images were acquired every 7.5 mins over 8 hrs using point visiting and a Coolsnap HQ (Photometrics) camera. TIRF live-cell images were acquired on a TE2000 microscope (Nikon), equipped with a perfect focus system, using an 100x/1.49 Apo TIRF objective and 488nm laser. TIRF images were acquired every 1.5 mins over 2 hrs using point visiting and a Cascade 512B EM CCD camera (Photometrics). Time projections and kymograph analyses, to show FA translocation, were generated using the ImageJ Z-projection function and MultipleKymograph plugin applied to stacks of time-lapse images (Epifluorescence Z-projection: 18 frames; Kymograph: 30 frames; TIRF Z-projection: Dimensionality of 80 x 1.5min frames reduced to 16 x 7.5 min frames). FA translocation speed was assessed using the Manual Tracking plugin in ImageJ and the period during which the FAs were tracked was determined to be the FA lifetime.

Fluorescence Recovery After Photobleaching

Cells expressing GFP-vinculin were plated on glass-bottom dishes, coated using 10µg/ml plasma fibronectin, 24-48 hours post-transfection, and imaged at 37°C in Ham's F12 medium supplemented with 2% FBS. FRAP was performed on an

Olympus inverted microscope (IX71) equipped with a 488-nm FRAP laser under the control of DeltaVisionRT software (Applied Precision) using a 100x/NA1.40 Plan Apo objective. Images were acquired every 3 secs for 180 secs post-photobleaching. 1.0µm-diameter regions of interest were selected and half-time of recovery ($t_{1/2}$) and mobile fraction were calculated using softWoRx FRAP photokinetic analysis software. The mobile fraction indicates the amount of a molecule that is freely mobile, whereas changes in the $t_{1/2}$ signify alterations in diffusion or binding to/release from an immobile substrate within a complex (e.g. integrins).

Interference reflection microscopy

Cells were plated on glass-bottom 24-well plates, coated using 10µg/ml plasma fibronectin and imaged at 37°C in Ham's F12 medium supplemented with 2% FBS. Time-lapse images were acquired on an Olympus inverted microscope (IX71) using DeltaVisionRT software (Applied Precision) and a 60x/NA1.42 Plan ApoN objective. Endogenous FAs were visualised by interference reflection using a Sedat Quad Filter Set 86000v2 (Excitation Filter: Cy5 (BP 640/20); Emission filter: Rh-TR-PE (BP 555/28)). Images were acquired every 15 mins over 8 hrs using point visiting and ImageJ used to analyse FA dynamics. All images were flatfield subtracted and thresholds were applied to define FA positions at each time point. Colocalisation of FAs between 15 min time points was calculated, by subtracting thresholded images, to determine levels of FA stability.

Flow cytometry

To detect levels of cell surface expression, cells were detached using trypsin/EDTA or enzyme-free Hanks' based cell dissociation buffer (Invitrogen) and washed with 0.1% BSA/0.1% sodium azide in PBS. Cells were incubated with primary antibody for 30 minutes at 4°C, washed three times, and incubated with Alexa Fluor 488- or Alexa Fluor 647-conjugated secondary antibody at 4°C for 30 minutes. Cells were analysed on a Beckman Coulter Cyan ADP.

To assess H0-binding, H0 was fluorescently-labelled using an Alexa Fluor 647 Antibody Labeling Kit (Invitrogen), according to manufacturer's instructions, and binding assessed by flow cytometry. Cells were detached using enzyme-free Hanks' based cell dissociation buffer (Invitrogen), washed with DMEM, held in suspension for 20 mins, and labelled in suspension with 10µg/ml Alexa Fluor 647-conjugated H0 for 60 mins. Cells were washed 3 times with PBS and fixed with 4% formaldehyde before analysis on a Beckman Coulter Cyan ADP

Recycling Assay Antibodies

The following antibodies were used to capture integrins for ELISA to monitor recycling: anti-mouse α5 (5H10-27), anti-mouse αV (RMV-7), anti-mouse β3 (2C9.G2), anti-human α5 (VC5), anti-human αV (L230) and anti-human β3 (B3A) antibodies..

Immunofluorescence and image analysis

For standard immunofluorescence, cells were plated on sterile fibronectin-coated coverslips in Ham's F12 medium supplemented with 2% FBS for 3 hrs at 37°C and 8% CO_2. Cells were fixed with 4% (wt/vol) paraformaldehyde, permeabilised for 4 min at RT with 0.5% (wt/vol) TritonX-100 in PBS^- and blocked (0.1% BSA/0.1% sodium azide in PBS^-). MEFs were stained for mouse α5 integrin (5H10-27) or mouse αV integrins (RMV-7; Cymbus). TIFs were stained for human α5 integrin (mab11) or human αV (L230) subunits or human αVβ3 heterodimer (LM609). Immunofluorescent images were acquired on an Olympus IX71 using DeltaVisionRT software, 60x/NA1.40 Plan Apo or 40x/NA0.85 Uplan Apo objectives and Coolsnap HQ camera. The same microscope settings were used to acquire all images within each experiment. The same ImageJ settings were applied for all conditions within a single experiment. Thresholds were applied to background subtracted images to produce overlay images to restrict analysis to FAs. Mask overlays were applied to background subtracted 16 bit images and the integrated density of integrin fluorescence in FAs determined.

Arf6 effector pull down assays

Active Arf6 was assessed as described previously (Santy, 2001). To determine steady-state Arf6 GTP-loading, cells were plated on 10μg/ml fibronectin in Ham's F12 medium with 2% FBS for 4 hrs prior to lysis. To test the role of syndecan-4 engagement on Arf6 activity, cycloheximide treated-cells were spread on the central cell-binding domain of fibronectin (Fn6-10) for 90 mins then stimulated with soluble heparin-binding domain-containing fragment of fibronectin (H0), or vehicle control, for 90 mins (as described previously (Bass, 2007a)) prior to lysis. Clarified lysates were incubated for 45 mins with GST-GGA3-bound Sepharose beads at 4˚C. Beads were washed 3 times in wash buffer (Santy, 2001) and eluted with SDS sample buffer. Levels of bound active Arf6 were detected by western. Total cell lysates were used to determine total Arf6 levels.

Migration on cell-derived matrices

Cell-derived matrices were generated by primary human fibroblasts as described previously (Bass, 2007b). $5x10^3$ cells were allowed to spread on the matrices in MEF medium for 4 hours prior to filming. Time-lapse images were acquired on an AS-MDW live-cell imaging system (Leica) as described above. Cell migration was tracked using the Manual Tracking plugin for ImageJ. Migration speed and relative displacement (total distance divided by final displacement after 17 hrs 40 mins migration) are shown. Tail retraction defect frequency was manually scored for >400 cells per condition.

SUPPLEMENTAL REFERENCES

Appierto, V., Villani, M.G., Cavadini, E., Gariboldi, M., De Cecco, L., Pierotti, M.A., Lambert, J.R., Reid, J., Tiberio, P., Colombo, N.*, et al.* (2007). Analysis of gene expression identifies PLAB as a mediator of the apoptotic activity of fenretinide in human ovarian cancer cells. Oncogene *26*, 3952-3962.
Gehlsen, K.R., Davis, G.E., and Sriramarao, P. (1992). Integrin expression in human melanoma cells with differing invasive and metastatic properties. Clin Exp Metastasis *10*, 111-120.
Kinoshita, E., Kinoshita-Kikuta, E., Takiyama, K., and Koike, T. (2006). Phosphate-binding tag, a new tool to visualize phosphorylated proteins. Mol Cell Proteomics *5*, 749-757.
Mostafavi-Pour, Z., Askari, J.A., Parkinson, S.J., Parker, P.J., Ng, T.T., and Humphries, M. (2003). Integrin-specific signaling pathways controlling focal adhesion formation and cell migration. J Cell Biol *161*, 155-167.
