# Supplementary figures and images for: Syndecan-4 Phosphorylation Is a Control Point for Integrin Recycling
Source: Dev Cell. 2013 Mar 11;24(5):472–85. doi: 10.1016/j.devcel.2013.01.027 (PMC3605578; doi:10.1016/j.devcel.2013.01.027)

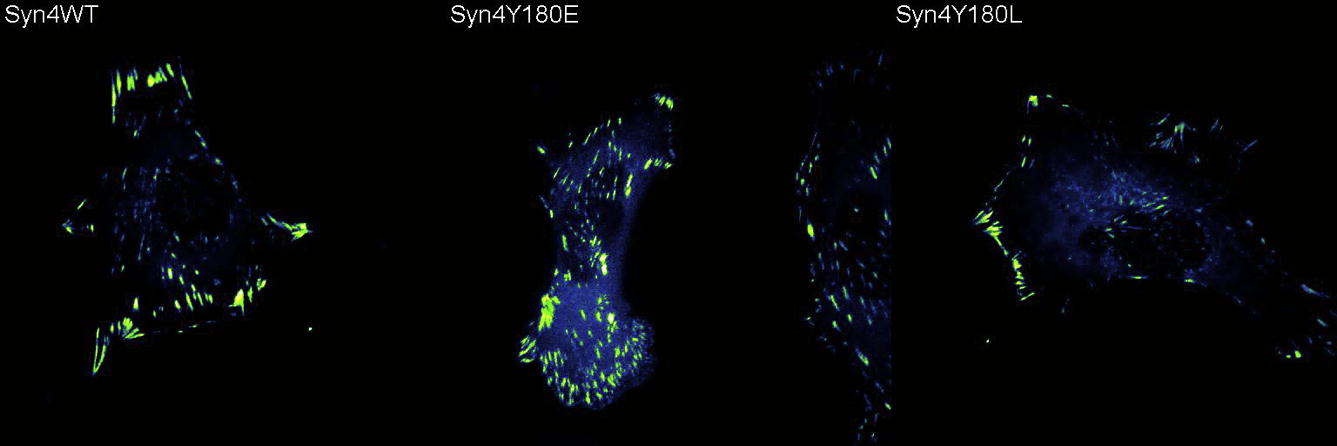

Supplement: Movie S2. Syndecan-4 Y180 Regulates FA Dynamics, Related to Figure 3 — Dynamics of GFP-vinculin-containing FAs in Syn4WT-, Syn4Y180E-, and Syn4Y180L-expressing cells (time = 8 hr). [file mmc3.jpg]

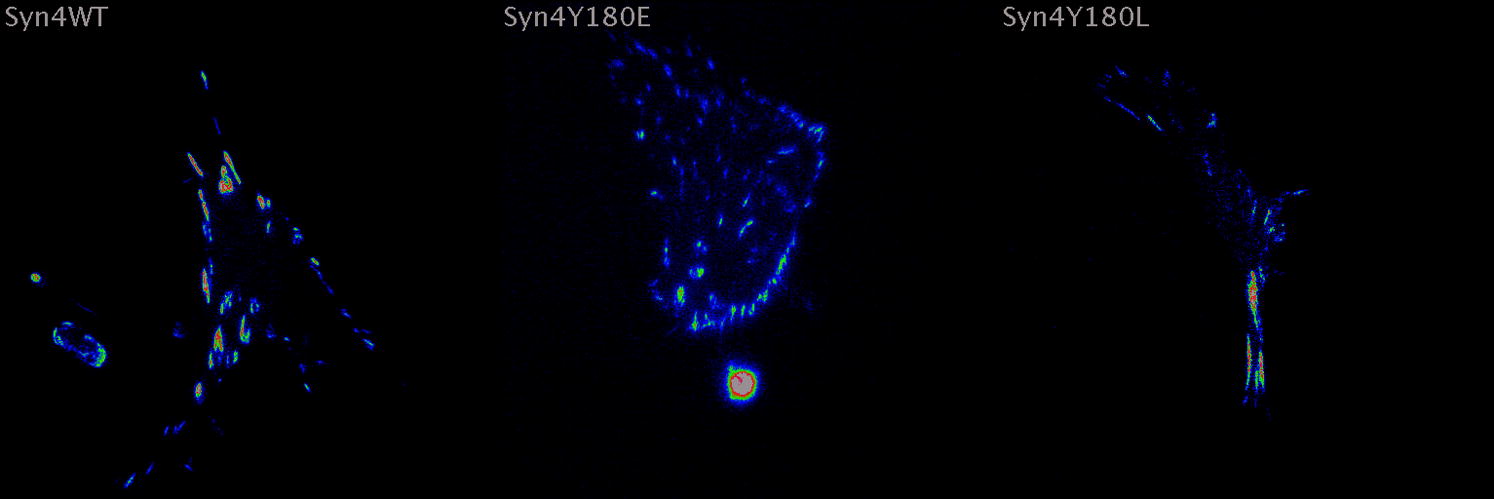

Supplement: Movie S3. S3 Arf6 Activity Regulates FA Dynamics, Related to Figure 7 — Dynamics of GFP-vinculin-containing FAs in Arf6WT-, Arf6T27N-, and Arf6T157A-expressing cells (time = 2 hr). [file mmc4.jpg]
